# miR-590-5p/Tiam1-mediated glucose metabolism promotes malignant evolution of pancreatic cancer by regulating SLC2A3 stability

**DOI:** 10.1186/s12935-023-03159-3

**Published:** 2023-11-28

**Authors:** Ying Liu, Aihua Jin, Xianglan Quan, Xionghu Shen, Houkun Zhou, Xingyu Zhao, Zhenhua Lin

**Affiliations:** 1 Central Laboratory, The Affiliated Hospital of Yanbian University, Yanji, 133000 People’s Republic of China; 2 Key Laboratory of Pathobiology (Yanbian University), State Ethnic Affairs Commission, Yanji, 133000 People’s Republic of China

**Keywords:** Tiam1, Pancreatic cancer, Metastasis, Glucose metabolic reprogramming, miR-590-5p

## Abstract

**Background:**

T lymphoma invasion and metastasis 1 (Tiam1) is a tumor related gene that specifically activates Rho-like GTPases Rac1 and plays a critical role in the progression of various malignancies. Glycolysis plays an important role in cancer progression, it is crucial for supplying energy and producing metabolic end products, which can maintain the survival of tumor cells. As yet, however, the mechanism of Tiam1 in glycolysis reprogramming of pancreatic cancer (PC) remains to be clarified. Here, we investigated the functional role of Tiam1 in PC cell proliferation, metastasis and glycolysis reprogramming. It is expected to provide a new direction for clinical treatment.

**Methods:**

The clinical relevance of Tiam1 was evaluated in 66 patients with PC, the effect of Tiam1 on cell proliferation was detected via 5-Ethynyl-2′-deoxyuridine (EdU) and colony formation. The ability of cell migration was detected by the wound healing and Transwell. Quantitative real time polymerase chain reaction (qRT-PCR) and luciferase reporter gene experiments clarify the regulatory relationship of miR-590-5p inhibiting Tiam1. Detection of the molecular mechanism of Tiam1 regulating glucose metabolism reprogramming in PC by glucose metabolism kit. RNA sequencing and Co-Immunoprecipitation (CoIP) have identified glucose transporter protein 3 (SLC2A3) as a key downstream target gene for miR-590-5p/Tiam1.

**Results:**

We found that Tiam1 expression increased in PC tissues and was associated with lymph node metastasis. The silencing or exogenous overexpression of Tiam1 significantly altered the proliferation, invasion, and angiogenesis of PC cells through glucose metabolism pathway. In addition, Tiam1 could interact with the crucial SLC2A3 and promote the evolution of PC in a SLC2A3-dependent manner. Moreover, miR-590-5p was found to exacerbate the PC cell proliferation, migration and invasion by targeting Tiam1. Furthermore, the reversing effects on proliferation, migration and invasion were found in PC cells with miR-590-5p/Tiam1 overexpression after applying glucose metabolism inhibition.

**Conclusions:**

Our findings demonstrate the critical role of Tiam1 in PC development and the miR-590-5p/Tiam1/SLC2A3 signaling pathway may serve as a target for new PC therapeutic strategies.

**Graphical Abstract:**

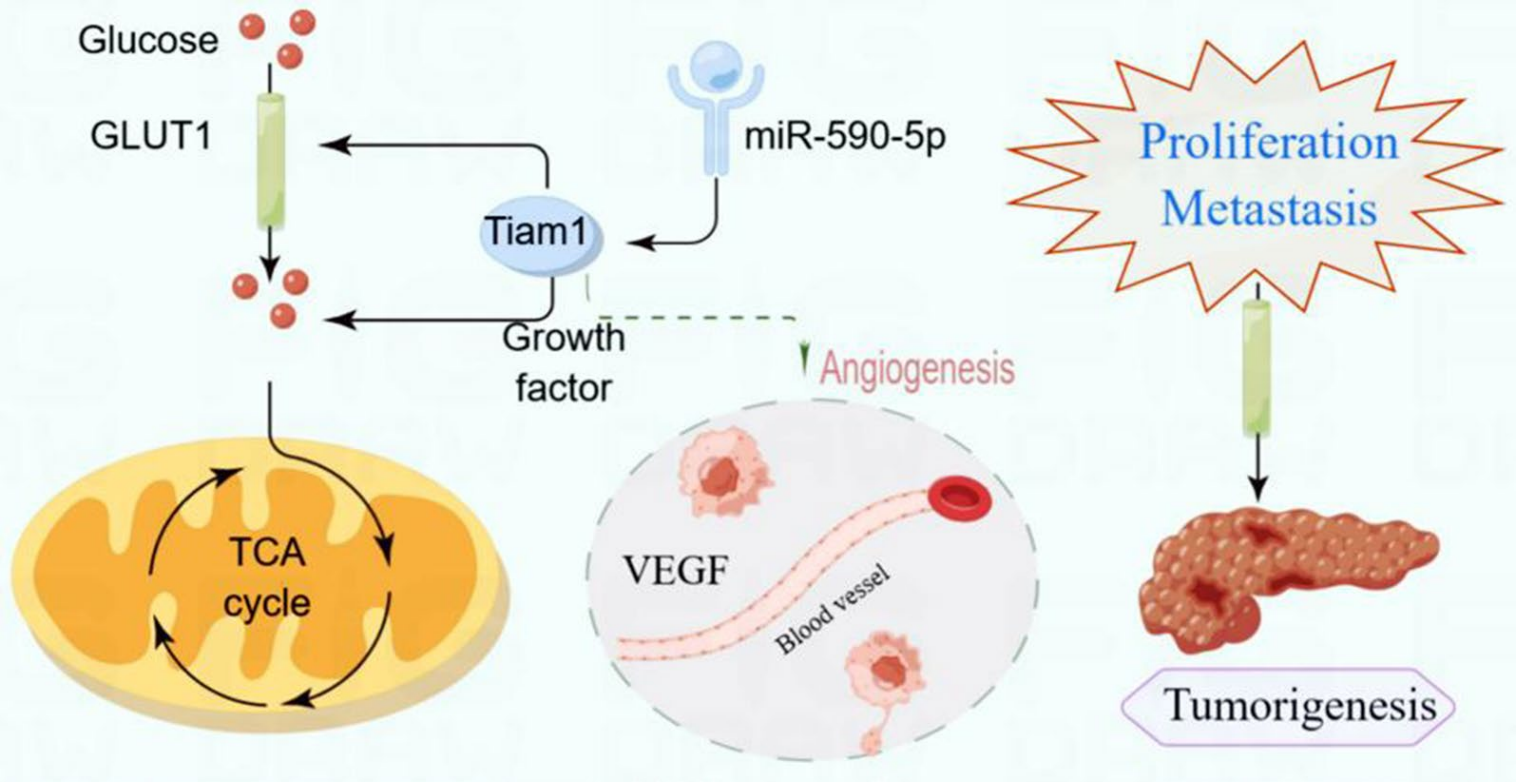

**Supplementary Information:**

The online version contains supplementary material available at 10.1186/s12935-023-03159-3.

## Background

Pancreatic cancer (PC) is a highly malignant tumor of the digestive system with a 5-year overall survival (OS) rate of approximately 9% [1]. The incidence of this deadly disease is increasing globally [2, 3]. Although the therapeutic intervention for PC has improved, tumor-related deaths caused by metastasis and recurrence remain a major challenge [4]. Once PC is diagnosed, the vast majority of cases show distant metastasis [5]. In addition, PC is less sensitive to radiotherapy, chemotherapy, and immunotherapy because of its intra-tumoral heterogeneity in cell metabolism, which is important for tumor growth and metastasis [6]. We previously found that the metabolic transition from oxidative phosphorylation to aerobic glycolysis is closely related to cell invasion [7]. Hyperglycemia induces metabolic reprogramming into a glycolytic phenotype and promotes epithelial–mesenchymal transition (EMT) via the Yes-associated protein (YAP)/Transcriptional coactivator with PDZ-binding motif (TAZ)-Hedgehog signaling axis in pancreatic cancer [8]. Therefore, further studies on activation of the crosstalk between aerobic glycolytic reprogramming and EMT in PC may provide novel strategies for treating tumors.

T lymphoma invasion and metastasis 1 (Tiam1) is located at chromosome 21q22.11 and contains 1591 amino acid residues with a molecular weight of 177 kDa [9]. As a guanine nucleotide exchange factor for the small GTPase Ras-related C3 botulinum toxin substrate 1 (Rac1), Tiam1 performs a wide variety of functions in cell adhesion, polarity, migration, transcription, and tumorigenesis [10, 11]. Tiam1 is specifically expressed in various malignant tumors and is closely related to the proliferation, migration, and invasion of tumor cells [12, 13]. Therefore, it is expected to be a biomarker for monitoring tumor progression and prognosis. Silencing Tiam1 reduces the proliferation and tumorigenicity of small cell lung cancer cells by promoting apoptosis [14]. Up-regulation of Tiam1 could promote proliferation and inhibit apoptosis of laryngeal squamous cell carcinoma (LSCC) after radiation both in vitro and in vivo [15]. We have previously found that Tiam1 is overexpressed in ovarian cancer (OC), gastric cancer (GC), and breast cancer (BC) and is associated with poor survival outcomes [16–18]. However, whether Tiam1 is involved in the mechanism of initiation and progression of PC is not clear.

In this study, we provide evidence that Tiam1 acts as an oncogene in PC, and that its oncogenic role correlates with its regulation of glucose metabolic reprogramming. Simultaneously, we aim to explore its upstream [microRNA (miR)-590-5p]/downstream (SLC2A3) roles in regulating glucose metabolism in PC and to identify Tiam1 as a valuable prognostic biomarker and therapeutic target.

## Methods

### Clinical specimens

We purchased tissue microarray from Outdo Biotech Co., Ltd., including PC (66 cases) with clinical features and normal pancreatic tissue (54 cases). The clinical pathological parameters include age, gender, tumor size, clinical staging, pathological grading, and lymph node metastasis. An informed consent form was signed before to collection, and all human tissue samples were obtained from accredited cooperative hospitals after consultation with pathology experts. The diagnosis and staging are based on the 8th edition manual of the United States Joint Commission on Cancer Staging.

### Cell culture and transfection

The human PC cell lines Patu8988, MIAPaca-2, Bxpc-3, and Panc-1 were obtained from the Typical Culture Collection Center (ATCC, Rockville, MD, USA). PC cells cultured in DMEM medium containing 1%Penicillin streptomycin and 10% FBS in a humid atmosphere containing 5% CO_2_ at 37 °C. For stably transfected PC cells, human Lenti shTiam1-GFP, Lenti Tiam1-GFP and control groups were packaged in HEK293T cells. After lentivirus transfection, purithromycin is used to kill PC cells that have not been transfected into the virus, thereby establishing stable expression PC cell line. For transient transfection, Tiam1 siRNA, and SLC2A3 siRNA (RIOBIO, China). Due to the knockout effect of siRNA, si-Tiam1#1, si-Tiam1#2, and si-SLC2A3#2 were significantly expressed in this study, which can be used for subsequent research. According to the manufacturer's instructions, PC cells and Lipofectamine 3000 (ThermoFisher, USA) were transfected with 30nM siRNA. All cell lines are identified by the supplier.

### Transwell assays

To verify the migration ability of cells, Transwell experiments were conducted for detection. 3 × 10^5^ MIAPaca-2 and Bxpc-3 cells were added to the upper chamber with 1% serum DMEM medium and 800 μl DMEM medium containing 200 μl FBS was added to the lower chamber. Unlike migration. The invision needs to apply matrix glue in the upper chamber 8 h in advance before applying cells. After 24 h, clean the bottom of the upper chamber twice with PBS, fix the cells with 4% paraformaldehyde for 5 min, clean again with DDW twice, and finally add 1 mL of hematoxylin to the lower chamber. Finally, place the upper chamber in the lower chamber for 30 min for staining. The migration cells were observed under microscope, the above experiments are repeated three times and the data were analyzed by Image J software.

### Luciferase reporter assay

The MIAPaca-2 and Bxpc-3 cells are digested and counted as 2 × 10^5^ cells were put into the 96 well plate when their density has fused to 80%. Co-transfect the reporter gene plasmid and transcription factor expression plasmid into cells, then purify the protein and utilize it to detect luciferase. Finally, add the substrate, check the luciferase activity, figure out the relative fluorescence intensity, and compare it to the empty control. Luciferase activity was measured using a Dual Luciferase Reporter Assay System (Promega, USA). Perform statistical analysis after repeating the experiment three times.

### Western blot (WB) analysis

RIPA Lysis Buffer (RLB, CWBIO, China) cell lysis solution, phosphatase inhibitors, and protease inhibitors were added for cell lysis after PC cells had been cleaned with phosphate-buffered saline (PBS). The sample's total protein concentration was ascertained using techniques like the Bradford, Lowry, or BCA determination. To transfer proteins from gel to membrane, prepare a 6–12% SDS-PAGE gel electrophoresis. Add an antibody after sealing, then incubate at 4 °C overnight. After secondary antibody treatment on the second day, protein bands were found using an enhanced chemiluminescence (ECL, Bio-rad, USA) kit. The above experiments were repeated three times and statistically analyzed through Image J software and prism 9.0 software.

### Coimmunoprecipitation (CoIP) and ubiquitination assay

PC cells expand to a size of over 90% before being lysed to produce a total protein solution. The PC cell supernatant is first treated with Tiam1 or SLC2A3 antibodies at 4 °C for an overnight period, and then for 6–8 h with magnetic bead complexes in a refrigerator. The protein antibody complex was isolated from the bulky magnetic beads the following day by washing and eliminating non-specific binding proteins. The WB experiment was then employed for detection. Simultaneously, ubiquitination tests were carried out using the aforementioned procedures after adding MG132 (Proteasome inhibitors, Santa Cruz Biotechnology) to PC cells for an hour, and were then verified using WB experiments. Perform statistical analysis after repeating the experiment three times.

### Immunohistochemistry (IHC)

After removing the tissue chip and placing it in an oven for 50 min, it was dewaxed with xylene. Place the tissue slices in heated sodium citrate buffer for antigen repair. Add 3% H_2_O_2_ dropwise to block endogenous peroxidase for 20 min, then block it and incubate it overnight at 4 °C. The next day, after cleaning, secondary antibody treatment was performed for 1 h, and DAB (double antibody, Zhong Shan Jin Qiao, China) was stained with tissue chips. Finally, two pathologists used a coupled scoring system, combining staining intensity and area range, to score all specimens in a blind manner. The scoring follows the previously described criteria [19]. 0–1 is negative (−), 2–4 is weakly positive (+), and 5–7 is moderately positive (++), ≥ 8 is strongly positive (+++). The tissue of ++ and +++ are considered to be strongly positive for indicator antibodies by Image J software, all the above experiments were conducted three times.

### Mouse xenograft model

This study was conducted with the approval of the Ethics Committee of Yanbian University School of Medicine. We obtained 4-week-old BALB/c nude mice from Beijing (Vital River Laboratory Animal Technology Co. Ltd., Beijing, China) and injected them subcutaneously with 100μ MIAPaca-2 and Bxpc-3 in PBS (3 × 10^6^ cells/mouse), 20 mice of different weights were randomly divided into 4 groups (5 in each group). After 8 weeks, the mice were euthanized, and the tumors were removed and weighed for further research and analysis. This experiment was completed in the SPF Animal Laboratory of the Animal Experimental Center of Yanbian University. This experiment was approved by the Animal Protection Committee of Yanbian University (SCXK (JI) 2017-0003) and was conducted in accordance with internationally recognized principles for the use and care of experimental animals.

### Statistical analysis

The data analysis was conducted using SPSS 26.0 software, GraphPad Prism 9.0 software, and Image J software to assess the correlation between Tiam1 expression and clinical pathological features in the study. The *t*-test of independent means is used for inter group comparison. Compare continuous data between groups using one-way ANOVA. All experiments were conducted in triplicate. Values with *p* < 0.05 are considered statistically significant.

## Results

### Tiam1 expression is increased and associated with poor prognosis of PC

**Fig. 1:**
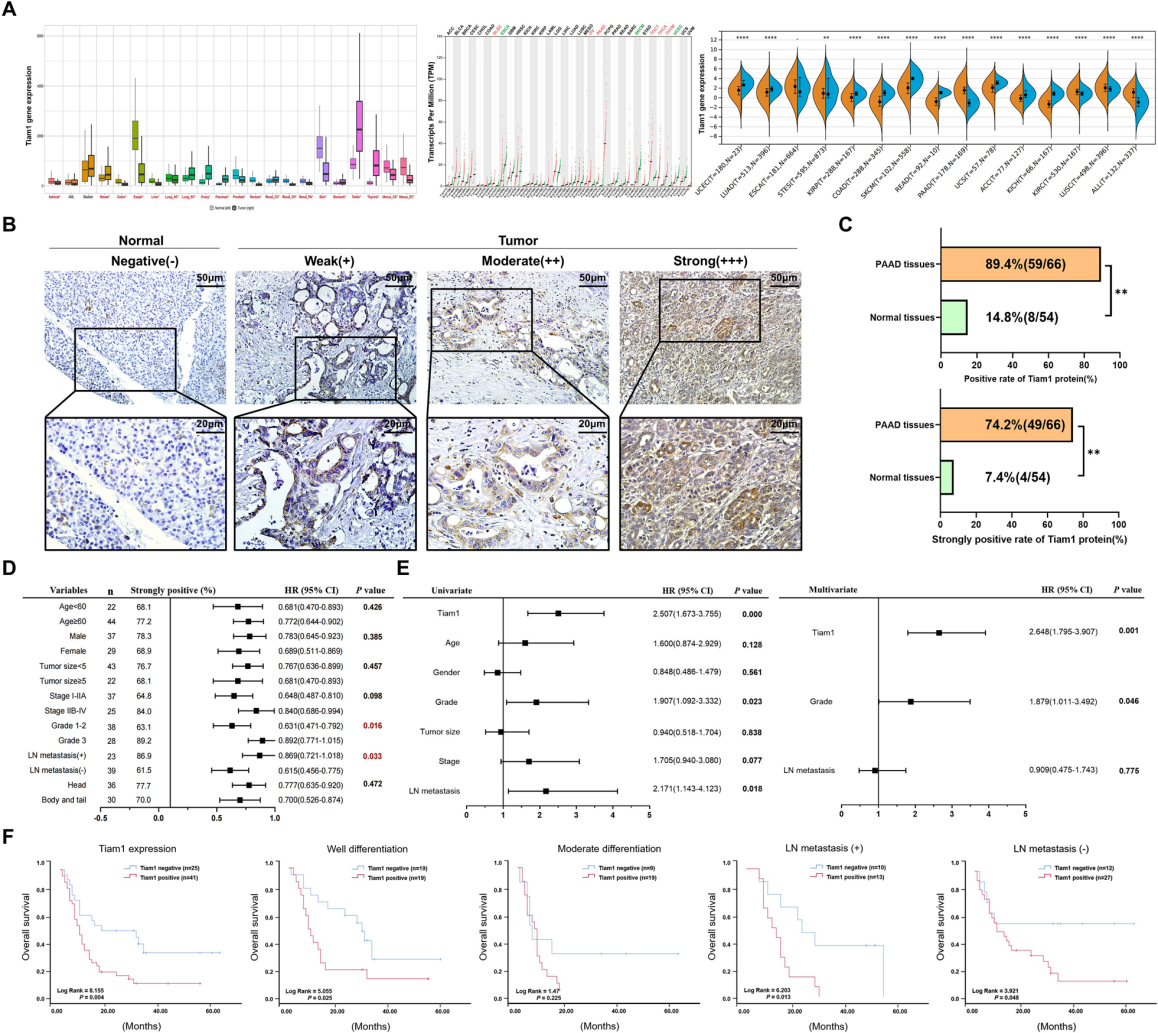
Tiam1 is overexpressed and associated with poor prognosis in pancreatic cancer (PC). **A** Expression of Tiam1 mRNA in normal and tumor tissues as indicated by the KM-plotter, Sangerbox, and GEPIA databases. **B**, **C** Immunohistochemistry results displaying strongly positive staining rates in PC and adjacent tissues at 200 × and 400 × magnification. **D** Correlation between Tiam1 expression and clinicopathological features of PC, represented by forest plots. *p < 0.05, **p < 0.01. **E** Forest plots depicting the results of univariate and multivariable logistic regression analyses. **F** Kaplan–Meier survival analysis using the KM plotter database to illustrate the impact of Tiam1 on overall survival (OS), differentiation, and lymphatic metastasis in patients with PC

To gain a deeper understanding of the Tiam1 in pancreatic cancer (PC), we analysed the expression of Tiam1 is highly expressed in PC tissues compared to that in adjacent normal pancreatic tissues according to the KM-plotter, GEPIA, and Sangerbox portals (Fig. 1a). IHC staining demonstrated a significant upregulation of Tiam1 expression in PC tissues in comparison to normal pancreatic tissues (Fig. 1b). 8 out of 54 normal tissues showed positive Tiam1 expression, with a positivity rate of 14.8%, while 59 out of 66 PC tissues exhibited positive Tiam1 expression, with a much higher positivity rate of 89.4% (*p* < 0.05). Furthermore, the strongly positive rate of Tiam1 expression in PC tissues (49/66, 74.2%) was significantly higher compared to that in non-tumor tissues (4/54, 7.4%, *p* < 0.05) (Fig. 1c). Additionally, our clinicopathological analysis revealed a positive correlation between Tiam1 expression and tumor grade (*p* = 0.016) as well as lymph node (LN) metastasis (*p* = 0.033), as illustrated in the forest plot (Fig. 1d). In addition, forest plots showed the results of univariate cox regression analysis, in which high expression of Tiam1 was related to patients’ grades [*p* = 0.023, hazard ratio (HR) = 1.907, 95% class interval (CI) = 1.092–3.332] and LN metastasis (*p* = 0.018, HR = 2.171, 95% CI = 1.143–4.123). Multivariate Cox regression analysis revealed that Tiam1 expression (*p* = 0.001, HR = 2.648, 95% CI = 1.795–3.907) and grade (*p* = 0.046, HR = 1.879, 95% CI = 1.011–3.492) were independent indicators of OS in PC (Fig. 1e). Kaplan–Meier analysis showed that OS rates were significantly higher in patients with Tiam1-negative phenotype than those with Tiam1-positive phenotype. Additionally, compared with Tiam1-negative patients, patients with a well-differentiated status (*p* = 0.025), negative LN metastasis (*p* = 0.048), and positive LN metastasis (*p* = 0.013) had significantly reduced OS. However, Tiam1-positive patients with moderate differentiation were not obvious (Fig. 1f). These findings indicate a significant association between high Tiam1 expression and poor prognosis in patients with PC.

### Tiam1 accelerates the proliferation of PC cells in vitro and in vivo

**Fig. 2:**
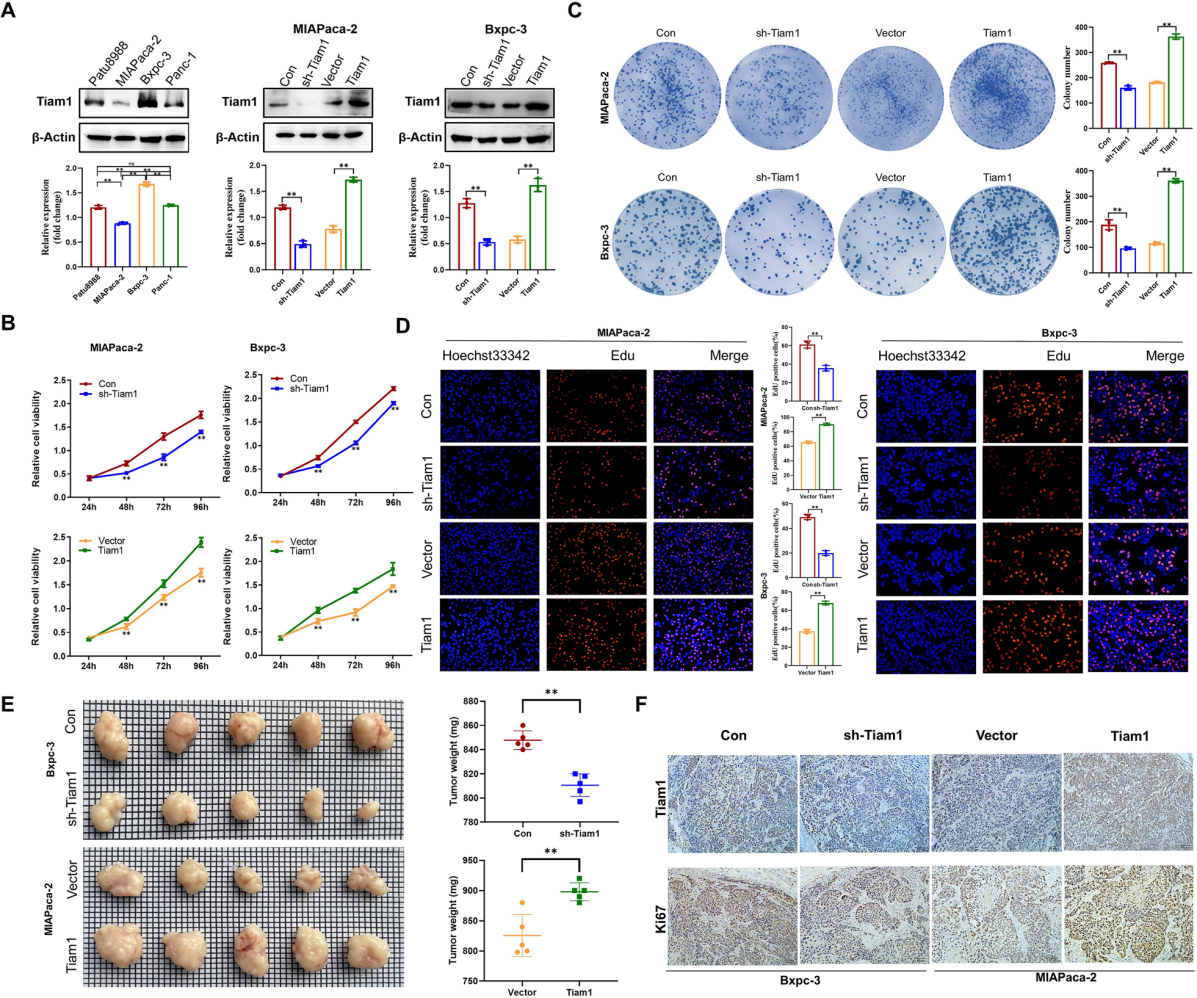
Tiam1 promotes the growth of pancreatic cancer (PC) cells in vitro and in vivo. **A** Tiam1 expression in Patu8988, MIAPaca-2, Bxpc-3, and Panc-1 cell lines, and successful generation of Tiam1 lentivirus-transfected MIAPaca-2 and Bxpc-3 cell lines analyzed by western blotting. **B**–**D** Proliferation of PC cells assessed through 3-[4,5-dimethylthiazol-2-yl]-2,5 diphenyl tetrazolium bromide (MTT), colony formation, and 5-ethynyl-2′-deoxyuridine (EdU) assays, displayed at 100 × magnification. **E** Tumor images formed by stably transfected cells injected into nude mice. Different groups of subcutaneously transplanted tumors were established. **F** Immunohistochemical staining of different groups depicting the expression of Tiam1 and Ki67 in PC tissues at 200 × magnification

WB analysis showed that Tiam1 was expressed in Patu8988, MIAPaca-2, Bxpc-3, and Panc-1 cell lines, with Bxpc-3 with significantly high expression and MIAPaca-2 with significantly low expression. Therefore, Bxpc-3 and MIAPaca-2 cells were selected for subsequent experiments. Both Bxpc-3 and MIAPaca-2 cells were stratified into four groups: control, sh-Tiam1, vector, and Tiam1 overexpression through lentiviral transfection. The transfection efficiency was assessed using WB analysis (Fig. 2a). Cell proliferation and clonogenicity were assessed using 3-(4,5-Dimethylthiazol-2-yl)-2,5-diphenyltetrazolium bromide (MTT), colony formation, and EdU assays. The findings demonstrated that sh-Tiam1 suppressed both cell growth and clonogenicity in PC, whereas Tiam1 overexpression had the opposite effect (Fig. 2b–d). Further validation was conducted in vivo experiments, providing evidence of Tiam1’s promoting role in tumor development. Knockdown of Bxpc-3 leads to a decrease in tumor size and weight, while overexpression of MIAPaca-2 enhances tumor size and weight. Consistent with in vitro experiments, IHC staining of xenograft tumor sections further showed that after Bxpc-3 knockdown, the expression levels of Tiam1 and Ki67 were downregulated in the sh-Tiam1 group, while after high expression of MIAPaca-2, the overexpression levels of Tiam1 and Ki67 were upregulated (Fig. 2e–f). Taken together, these results demonstrate the significant oncogenic roles of Tiam1 in PC.

### Tiam1 regulates metastasis of PC cells through EMT

**Fig. 3:**
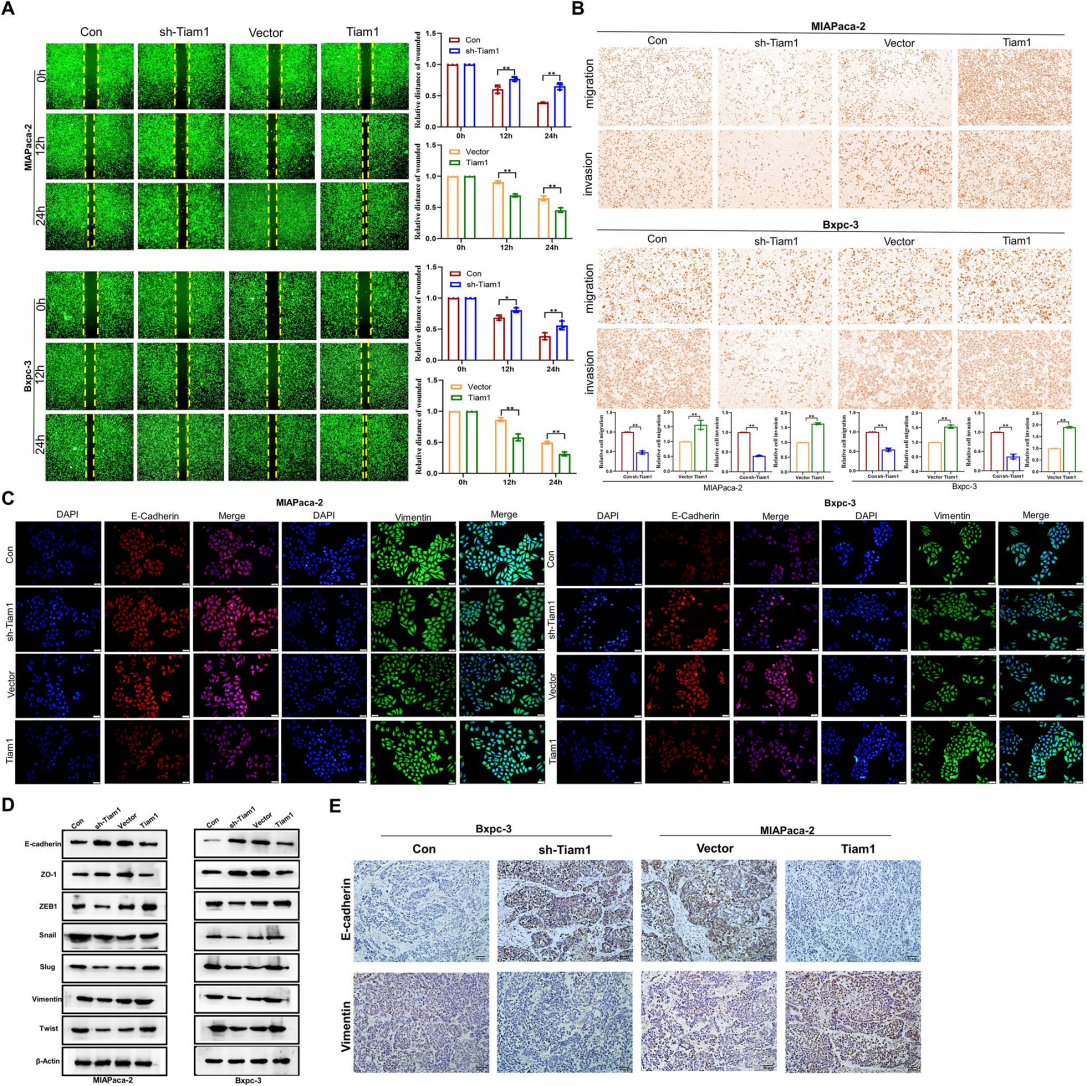
Tiam1 promotes pancreatic cancer (PC) metastasis via epithelial–mesenchymal transition (EMT) in vitro and in vivo. **A**, **B** Wound healing and Transwell assays showed horizontal and vertical migration of Tiam1 in PC cells. **C** Immunofluorescence staining showed the expression of EMT markers in PC cells (100 ×). **D** Western blot analysis EMT markers. β-actin was used as a loading control. **E** Immunohistochemical staining showed E-cadherin and vimentin expression in tissues of different groups (200 ×)

To investigate the functional relevance of Tiam1 in the metastasis of PC cells, we observed that the knockdown of Tiam1 significantly inhibited both lateral and longitudinal migration of PC cells compared to the control group. These findings were demonstrated through wound healing and Transwell assays. Conversely, opposite results were observed in Tiam1-overexpressed cells (Fig. 3a, b). Moreover, we analyzed changes in the expression of EMT markers in Tiam1-overexpressed or Tiam1-knockdown PC cells by IF (Immunofluorescence) and WB. As expected, the expression of E-cadherin (epithelial marker) was increased, and the level of vimentin (mesenchymal marker) was decreased in Tiam1-knockdown cells. The overexpression of Tiam1 had an opposing effect (Fig. 3c, d). At the same time, IHC stained tumor sections with E-cadherin and vimentin to quantitatively evaluate EMT related changes. After Bxpc-3 silencing, the expression level of E-cadherin increased and vimentin decreased. After high expression of MIAPaca-2, the expression level of E-cadherin decreased and vimentin increased. The results showed the same trend as in vitro cell experiments (Fig. 3e). Taken together, these data imply that Tiam1 expression is closely associated with EMT progression in PC.

### Tiam1 promotes the ability of angiogenesis in PC cells

**Fig. 4:**
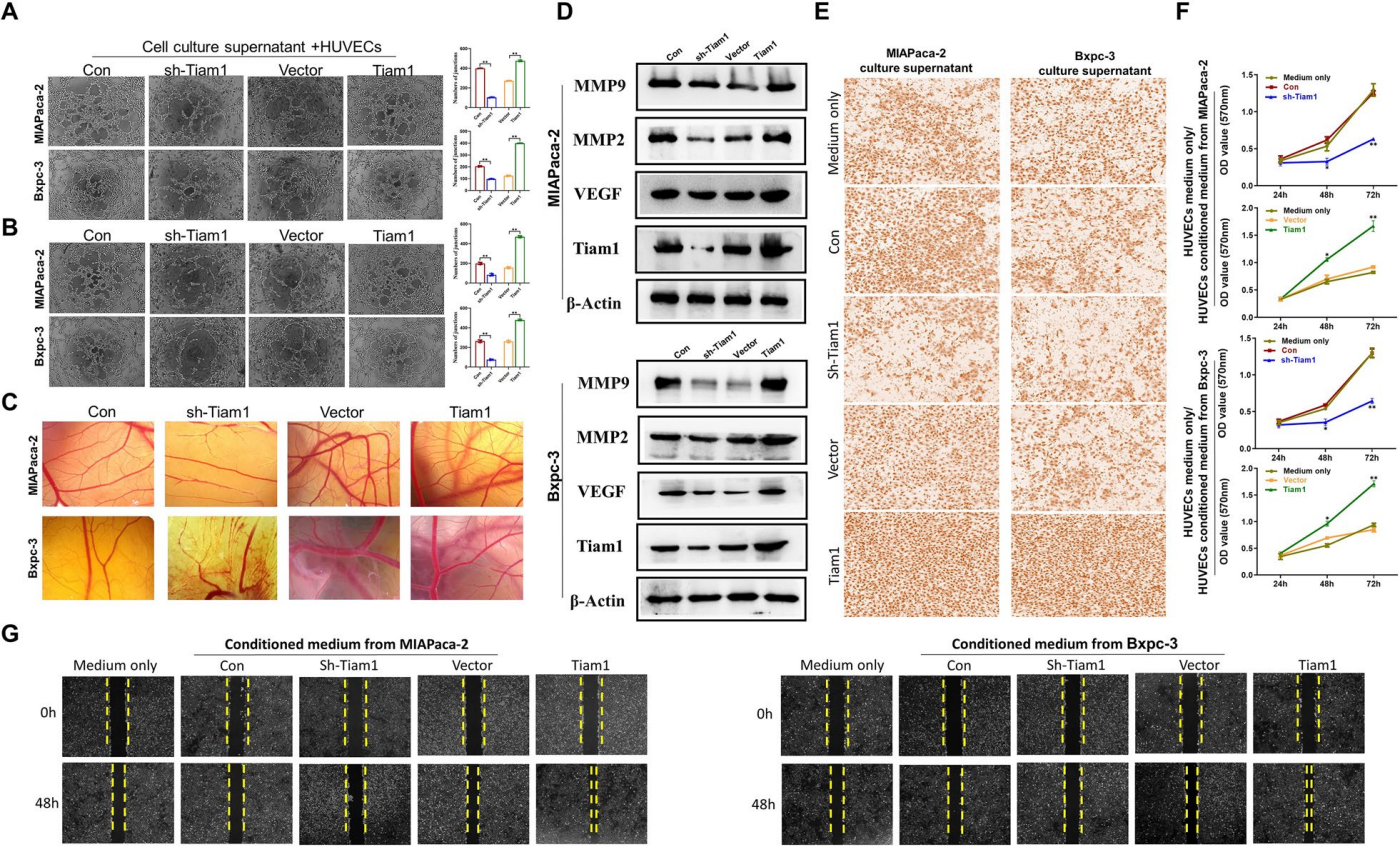
Tiam1 promotes angiogenesis in pancreatic cancer (PC) cells and enhances the growth and migration of Human Umbilical Vein Endothelial Cells (HUVECs). **A** Matrigel tube formation assay depicting microtubule formation in HUVECs cultured with the supernatants of PC cells. **B** Vasculogenic mimicry assay evaluating the ability of vascular mimicry in the four groups of PC cells. **C** Chick chorioallantoic membrane (CAM) assay assessing the effect of Tiam1 on ex vivo angiogenesis. **D** Western blot analysis revealing the expression of matrix metalloproteinase MMP9, MMP2, vascular endothelial growth factor (VEGF), and Tiam1 in the four groups of PC cells. **E**–**G** Migration of HUVECs cultured with medium and the supernatants of PC cells was evaluated through Transwell and wound healing assays, while the proliferation of HUVECs was detected by 3-[4,5-dimethylthiazol-2-yl]-2,5 diphenyl tetrazolium bromide (MTT) assay. In all panels, data are presented as mean ± standard deviation (SD). *p < 0.05, **p < 0.01

Vasculogenic mimicry and Matrigel tube formation experiments showed that sh-Tiam1 cells' capacity for vascular mimicry and microtubule production was reduced, but Tiam1 overexpression cells' capacity was increased (Fig. 4a, b). By using the chick embryo chorioallantoic membrane (CAM) experiment to conduct additional research on the effect of Tiam1 on angiogenesis in vivo, it was discovered that vascular rupture occurs when Tiam1 cells are knocked down. On the other hand, enhanced Tiam1 expression boosted PC cells' angiogenic potential and raised the quantity of newly created blood vessels (Fig. 4c). WB analysis demonstrated that Tiam1 depletion resulted in a downregulation of the expression of angiogenic markers (Fig. 4d). These findings confirm that Tiam1 may indeed play pro-angiogenic roles in PC. Simultaneously, we investigated whether Tiam1 affects the proliferation and migration of human umbilical vein endothelial cells (HUVEC) cells. HUVECs cells are co-cultured with PC cell supernatant that has Tiam1 expressed differently, and the results are validated using Transwell, MTT, and wound healing tests (Fig. 4e–g). These collective findings strongly suggest that Tiam1 might modulates the metastatic behavior of PC cells by regulating angiogenesis.

### Tiam1 regulates reprogramming of glucose metabolism in PC cells

**Fig. 5:**
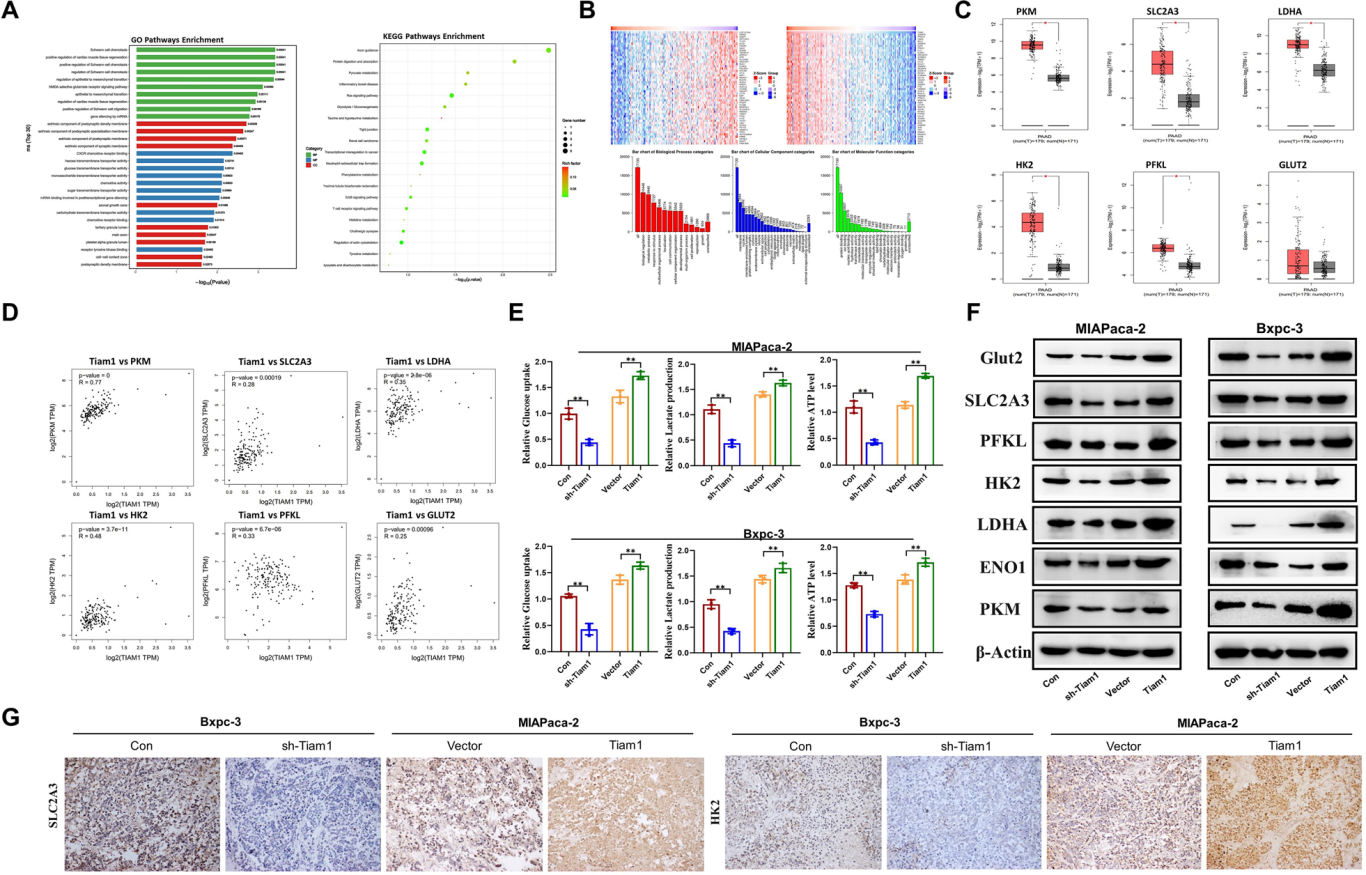
Tiam1 regulates reprogramming of glucose metabolism in pancreatic cancer (PC) cells. **A** RNA sequencing analysis indicated significant enrichment of Tiam1 in the biological process of glucose metabolism in PC. **B** Genes interacting with Tiam1 and the associated biological processes (false discovery rate, FDR < 0.01). **C**, **D** Expression of glucose metabolism-related proteins in PC, with a significant positive correlation observed between these proteins and Tiam1 (*p* < 0.05). **E** Tiam1 knockdown and Tiam1 overexpression affect glucose uptake, lactate production, and ATP expression levels in PC cells. **F** Western blot analysis depicting the expression of glucose metabolism-related markers in PC cells, with β-actin serving as a loading control. **G** Immunohistochemical staining illustrating SLC2A3 and HK2 expression in tissues from different groups at 200 × magnification

**Fig. 6:**
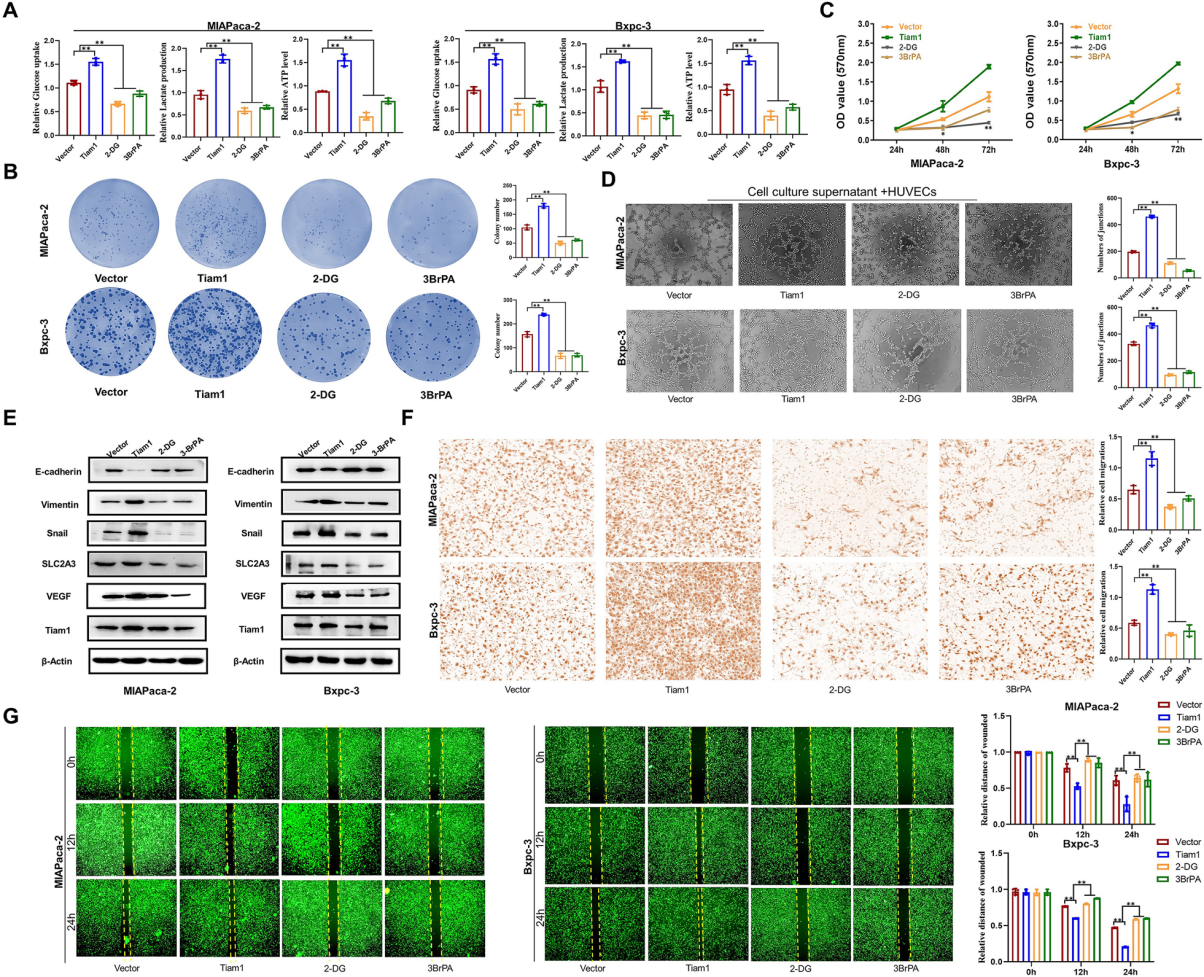
Inhibition of Tiam1 expression in pancreatic cancer (PC) by the inhibitors of glucose metabolism. **A** Glucose uptake, lactate production, and ATP levels were measured in PC cell lines. **B** Colony formation assay indicated that 2-deoxy-d-glucose (2-DG) or 3-bromopyruvate (3BrPA) inhibited Tiam1 overexpression-induced cell proliferation. **C** 3-[4,5-Dimethylthiazol-2-yl]-2,5 diphenyl tetrazolium bromide (MTT) and **D** Matrigel tube formation assays. **E** Western blotting. **F** Transwell assay. **G** Wound healing assay. In all panels, data are presented as the mean ± standard deviation (SD). *p < 0.05, **p < 0.01

Glycolysis represents a central feature of metabolic reprogramming in cancer and serves as a metabolic hallmark of invasive cancers [22]. However, the precise metabolic mechanism is unknown. We noticed by RNA sequencing analysis that Tiam1 was significantly enriched in the biological process of glucose metabolism in PC (Fig. 5a). Likewise, an analysis of a public database revealed a close association between Tiam1 and the regulation of metabolic processes in PC. Tiam1 exhibited a significant positive correlation with markers related to glucose metabolism, which were highly expressed in PC (Fig. 5b–d). Hence, we started investigating whether Tiam1 controls the glycolytic nature of PC cells. In MIAPaca-2 and Bxpc-3 cells, the knockdown of Tiam1 resulted in decreased glucose uptake, lactate production, and adenosine triphosphate (ATP) levels, while the opposite effects were observed in Tiam1-overexpressed cells (Fig. 5e). In line with these findings, WB analyses demonstrated that the glucose transporter *SLC2A3* and glycolytic enzymes *PFKL, HK2, LDHA, ENO1,* and *PKM* exhibited reduced expression in response to Tiam1 knockdown, whereas their expression was elevated upon Tiam1 overexpression. Consistent with the above results, IHC results also showed that after Bxpc-3 silencing treatment of cells, the expression levels of glucose metabolism related proteins SLC2A3 and HK2 were also reduced. After high expression of MIAPaca-2 in cells, the expression levels of glucose metabolism related proteins SLC2A3 and HK2 were increased (Fig. 5f, g). We subsequently investigated whether glycolysis plays a role in the Tiam1-mediated malignant phenotype of PC cells. The use of glycolysis inhibitors, namely 2-deoxy-d-glucose (2-DG) and 3-bromopyruvate (3BrPA), led to a significant reduction in glucose uptake, lactate production, and ATP levels in PC cells (Fig. 6a). Consistent with these results, the application of 2-DG and 3BrPA almost entirely abolished the effects of Tiam1 on clonogenesis, proliferation, and angiogenesis in PC cells (Fig. 6b–d, Additional file 1: Fig. S1A). The addition of 2-DG and 3BrPA significantly inhibited EMT biomarkers expression level of PC cells (Fig. 6e). In addition, wound healing and Transwell experiments showed that 2-DG and 3BrPA inhibited vertical and horizontal migration of PC cells (Fig. 6f, g, Additional file 1: Fig. S1B). In summary, these data indicate that Tiam1 participates in regulating PC cell proliferation and metastasis through aerobic glycolysis.

### Tiam1 exerts oncogenic effects through regulating the stability of SLC2A3 in PC

**Fig. 7:**
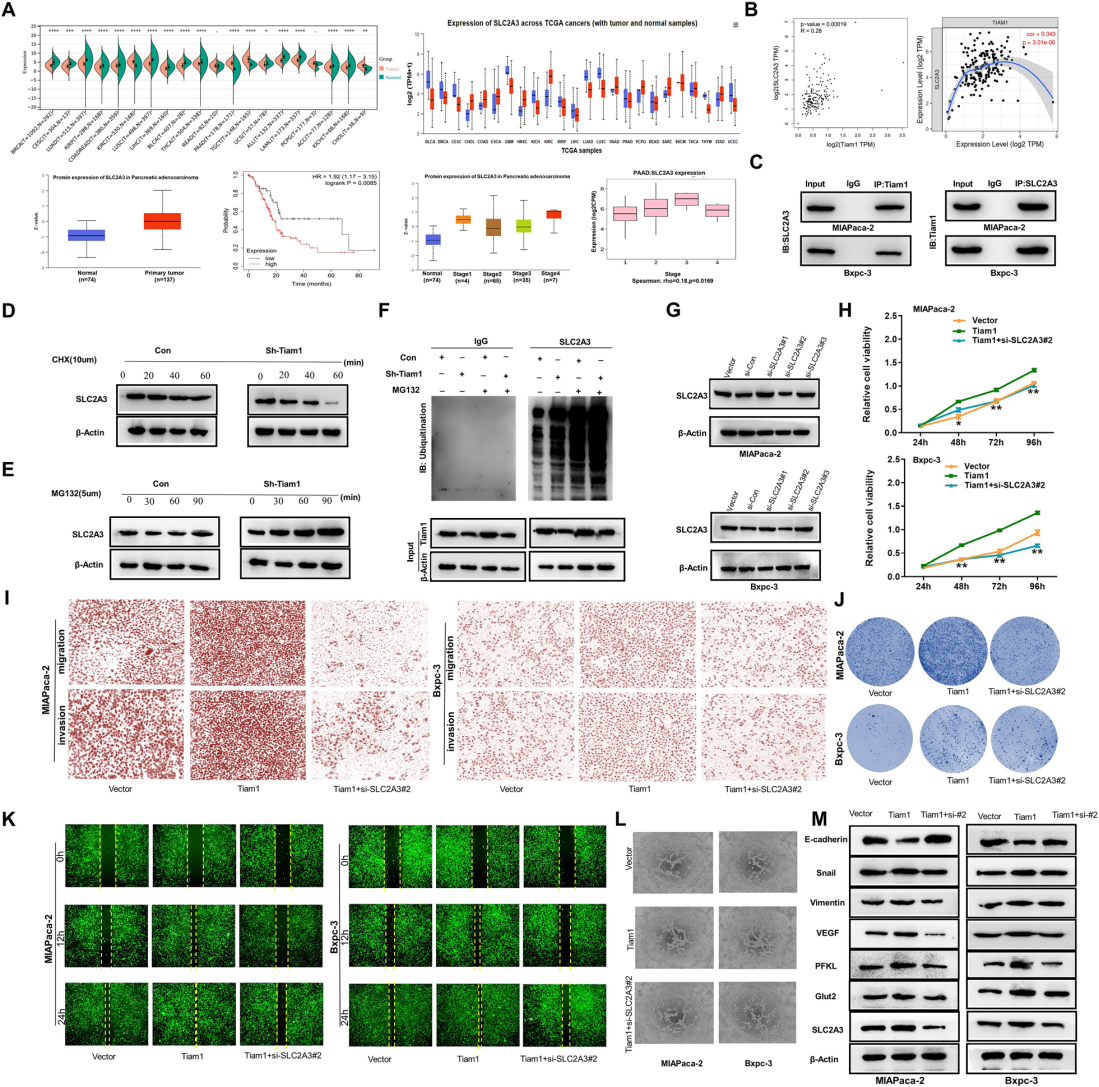
Tiam1 exerts oncogenic effects through regulating ubiquitination of SLC2A3. **A**, **B** UALCAN, Kaplan–Meier Plotter, and Sangerbox showed high expression of SLC2A3 in pancreatic cancer (PC). GEPIA2 and TIMER2 found a significant positive correlation between SLC2A3 and Tiam1 in PC tissues (*p* < 0.05). **C** Coimmunoprecipitation further verified the combination between Tiam1 and SLC2A3. **D**,** E** PC cells were treated with 10 µM cycloheximide (CHX) 5 µM MG132 for the indicated time. SLC2A3 expression was detected by western blotting. **F** Ubiquitination of SLC2A3 was analyzed by western blotting. **G** Screening for expression of si-SLC2A3#2 by western blotting for subsequent experiments. **H** 3-[4,5-Dimethylthiazol-2-yl]-2,5 diphenyl tetrazolium bromide (MTT) assay.** I** Migration and invasion assay.** J** Colony formation assay.** K** Wound healing assay.** L** Matrigel tube formation assay. **M** Western blotting

UALCAN, Kaplan–Meier plotter, and Sangerbox analyses showed high expression of SLC2A3 in PC. High SLC2A3 expression was closely associated with poor prognosis and pathological staging of PC patients (Fig. 7a, *p* < 0.05). GEPIA2 and TIMER2 also showed a significant positive correlation between SLC2A3 and Tiam1 expression in PC tissues (Fig. 7b, p < 0.05). Coimmunoprecipitation analysis further confirmed the binding of Tiam1 and SLC2A3 (Fig. 7c). The differential expression of Tiam1 had an impact on the protein levels of SLC2A3 but did not affect its mRNA expression, suggesting that Tiam1 post-translationally regulates SLC2A3 expression in PC cells. To further investigate this regulation, PC cells were treated with cycloheximide (CHX) to inhibit de novo protein synthesis. This treatment led to a significantly shorter half-life of SLC2A3 in shTiam1 cells compared to the control group (Fig. 7d). Stabilization of SLC2A3 was achieved by inhibiting protein degradation using MG132 in sh-Tiam1 cells (Fig. 7e). Following the ubiquitination assay, we hypothesized that Tiam1 induces SLC2A3 stabilization by inhibiting ubiquitination (Fig. 7f). WB analysis revealed that the expression of si-SLC2A3#2 was significantly reduced in PC, and it was selected for subsequent experiments (Fig. 7g). si-SLC2A3#2 significantly mitigated Tiam1 overexpression-induced glycolysis, as well as the proliferation, metastasis, angiogenesis, and epithelial-mesenchymal transition (EMT) markers of PC cells (Fig. 7h–m). These results indicate that Tiam1/SLC2A3 knockdown inhibits tumorigenesis in PC.

### miR-590-5p/Tiam1 regulates reprogramming of glucose metabolism and tumor metastasis in PC

**Fig. 8:**
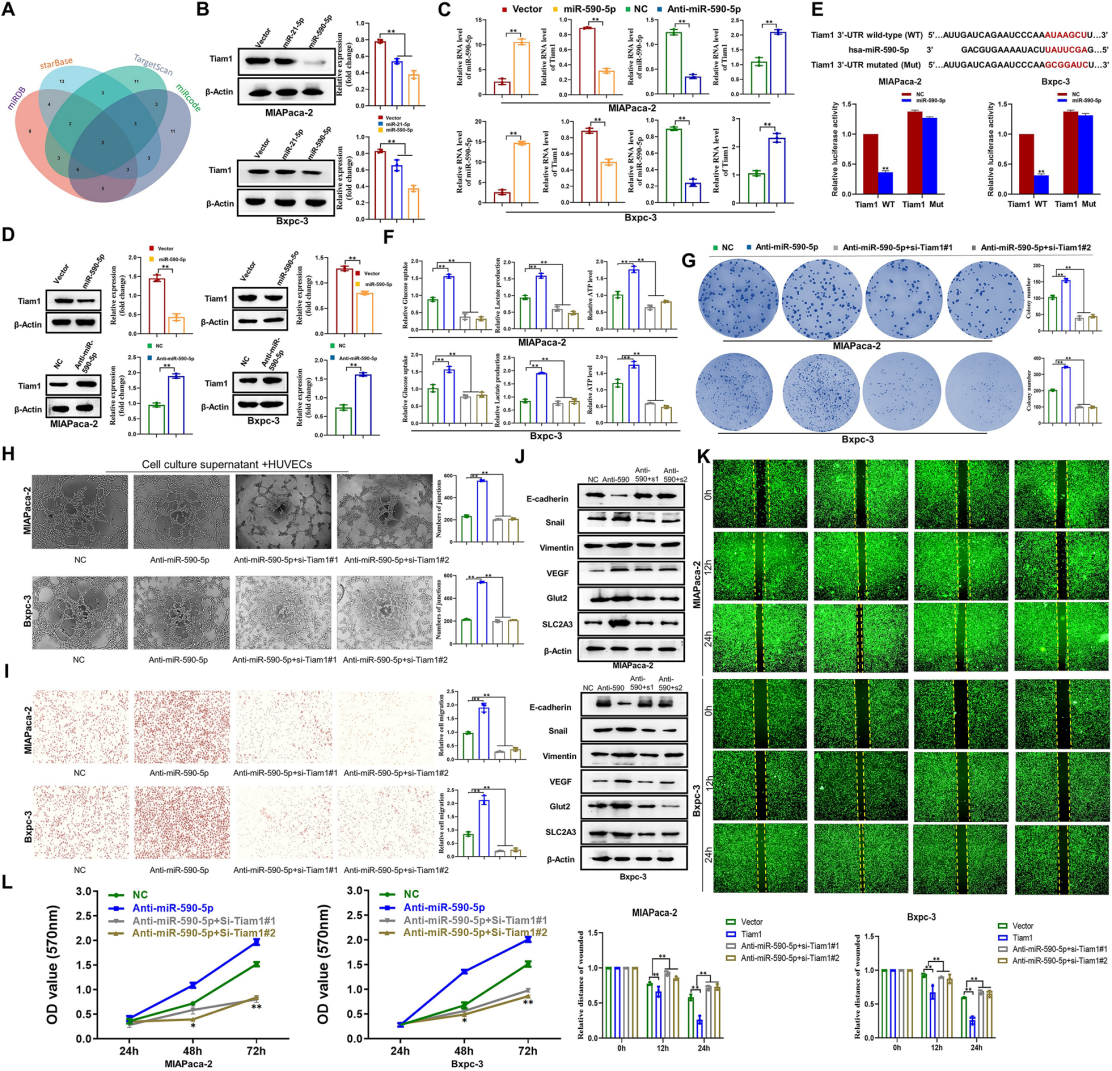
miR-590-5p/Tiam1 regulates pancreatic cancer (PC) progression. **A** Venn diagrams of four independent databases revealed possible upstream regulators of Tiam1.** B** Western blot showed the effect of miR-21-5p or miR-590-5p on Tiam1 expression in PC cells. **C**, **D** Quantitative real-time polymerase chain reaction and Western blot analysis displaying the mRNA and protein expression of Tiam1 following transfection of miR-590-5p and anti-miR-590-5p. **E** The binding sites of 3′-UTR and miR-590-5p. Dual luciferase assay to confirm the binding sites. **F** Glucose uptake, lactate production, and ATP levels. **G** Colony formation assay. **H** Matrigel tube formation assay. **I** Transwell assay. **J** Western blot analysis. **K** Wound healing assay. **L** 3-[4,5-Dimethylthiazol-2-yl]-2,5 diphenyl tetrazolium bromide (MTT) assay

miRNAs play an important role in the development and progression of tumors including their involvement in biological processes such as tumor cell growth, metabolism, and stress response [23]. Subsequently, we investigated the upstream regulatory mechanisms governing Tiam1 by utilizing four target prediction programs (miRDB, starBase, miRcode, and TargetScan) to assess the likelihood of functional binding sites. As a result, miR-21-5p and miR-590-5p were identified as potential upstream regulators (Fig. 8a). We conducted WB to evaluate the impact of these miRNAs on Tiam1 expression, and based on the results, miR-590-5p was chosen for further analysis (Fig. 8b). Tiam1 expression was discovered to be suppressed by miR-590-5p at both the mRNA and protein levels, whereas miR-590-5p inhibition increased Tiam1 expression in PC cells (Fig. 8c, d). A dual luciferase reporter test was carried out to verify miR-590-5p's sensitivity to the 3′UTR of Tiam1 mRNA. As shown in Fig. 8e, miR-590-5p reduced the luciferase activity of Tiam1 wild-type (WT) 3′-UTR but had no effect on the mutated (MUT) 3′-UTR, in which the binding sites of miR-590-5p were altered. These findings collectively suggest that miR-590-5p inhibits Tiam1 expression by directly targeting its 3′-UTR in PC cells. MiR-590-5p inhibitors were used to determine whether miR-590-5p influences glycolysis and metastasis by reducing Tiam1 expression in PC cells. Anti-miR-590-5p significantly increased glucose uptake, lactate production, and ATP levels of PC cells (Fig. 8f). These effects were counteracted by the use of si-RNA#1/2 targeting Tiam1 in cells transfected with anti-miR-590-5p. Additionally, an increase in PC cell proliferation, angiogenesis, EMT, and migration was seen after anti-miR-590-5p transfection. However, the addition of si-Tiam1#1/2 in cells transfected with anti-miR-590-5p lessened these effects (Fig. 8g–l, Additional file 1: Fig. S2). These results strongly indicate that miR-590-5p mediates glycolysis and tumor metastasis by directly targeting Tiam1 in PC cells.

**Discussion**

In this study, we observed that Tiam1 was frequently overexpressed in PC tissues and was associated with poor prognosis in patients with PC. Meanwhile, the xenograft tumor model experiment further verified that silencing Tiam1 of Bxpc-3 cell resulted in a corresponding decrease in tumor tissue weight. The IHC staining of tumor tissue inhibited the processes of EMT, Ki67 and glucose metabolism, providing better validation for in vitro experiments. The miR-590-5p–Tiam1 axis accelerated proliferation, migration, invasion, EMT, and angiogenesis of PC cells by enhancing aerobic glycolysis. Mechanistically, Tiam1 upregulated SLC2A3 expression by preventing its ubiquitin-mediated proteolysis via direct binding to SLC2A3.

Tiam1 is a Rac1-specific guanine nucleotide exchange factor that mediates the exchange of GDP for GTP [24]. Tiam1 regulates the Rac signaling pathways that influence cell shape, migration, adhesion, growth, endocytosis, and polarity [25, 26]. Tiam1 is frequently overexpressed in various types of cancer including breast [27], prostate [28], colorectal [29] and cervical [30] cancers. Our study showed that Tiam1 was significantly overexpressed in PC tissues, and elevated expression of Tiam1 predicted poor prognosis of patients with PC, highlighting its potential role as a new promising biomarker for PC prognosis. Notably, frequent increases in Tiam1 in various types of tumors affect multiple cellular processes such as proliferation, adhesion, migration, stemness, and infiltration [31, 32]. Tiam1 is highly expressed in thyroid cancer, and deletion of Tiam1 can inhibit the proliferation and metastasis of thyroid cancer cells [26]. Overexpression of Tiam1 can restore proliferation and invasion of LSCC cells [15].

As a crucial hallmark of cancer, glucose metabolic reprogramming has been implicated in the malignant transformation of cancers [33, 34]. To adapt to environmental pressures or to provide more ATP to maintain rapid growth, tumor cells prefer anaerobic glycolysis, even under normal oxygen conditions; this is called the Warburg effect [35, 36]. Glucose-dependent membrane transporter proteins mediate transmembrane diffusion into cells, with over 10 members of the glucose membrane transporter family [37]. Among them, GLUT3 is widely expressed in human tissues and organs and is the main transmembrane transporter of glucose [38]. Aerobic glycolysis in malignant tumors is abnormally active and consumes large amounts of glucose. Increased glycolysis permits allows rapid proliferation, chemoresistance, EMT, and metastasis of tumor cells [39, 40]. In this study, a significant decrease in glycolytic activity was observed in Tiam1-depleted PC cells, accompanied by inhibition of cell proliferation, migration, invasion, and angiogenesis. Moreover, Tiam1 interacted with SLC2A3, thereby regulating PC malignancy.

Multiple miRNAs are involved in aerobic glycolysis in tumor cells [41]. The involvement of miRNAs in regulating the Warburg effect can be divided into promoting and inhibiting effects that regulate the expression of various key enzymes [42]. In this study, miR-590-5p played an important role in aerobic glycolysis by targeting the 3′-UTR of Tiam1, reducing its expression leading to decreases in glucose consumption, lactate production, and ATP production, thereby inhibiting glycolysis. miR-590-5p is involved in many important cellular biological processes, specifically tumor-related signaling pathways [43]. The upregulation of miR-590-5p inhibits migration and invasion of MGC-803 and HGC-27 gastric cancer cells, and serum markers of miR-590-5p in exosomes have potential significance for an early detection of gastric cancer [44]. miR-590-5p promotes cisplatin resistance in ovarian cancer by negatively regulating hMSH2, which may serve as a therapeutic target for cisplatin resistant ovarian cancer [45]. Consistent with the above findings, we observed that miR-590-5p/Tiam1 regulated the molecular mechanism of glucose metabolic reprogramming and induced PC cell proliferation and metastasis. Therefore, miR-590-5p inhibited the malignant behavior of PC cells by inhibiting Tiam1 expression. In addition, co-expression of anti-miR-590-5p and si-Tiam1 largely reversed the tumor-promoting effect.

This study reveals the important role of Tiam1 in the development of PC, providing clues for the development of new treatment strategies and enabling better implementation of personalized healthcare. It can improve the early diagnosis rate of cancer, make treatment more successful, provide a better monitor treatment progress and make adjustments as needed. At the same time, there is a lack of understanding of the mechanism of the interaction between Tiam1 and SLC2A3 in our study. This means that although research has found them to be related to some extent, the exact molecular mechanism is still unclear. This may require further experiments and deeper research to clarify.

## Conclusions

In summary, our research reveals miR-590-5p/Tiam1/SLC2A3-mediated glycolytic reprogramming promotes PC progression for the first time. The miR-590-5p/Tiam1/SLC2A3 signaling pathway may be a new therapeutic target for treating PC. This study has improved our understanding of glucose metabolism in cancer cells and provided new ideas and directions for clinical treatment of cancer.

### Supplementary Information

**Additional file 1. Fig. S1**: Inhibition of Tiam1 expression in PC by glucose metabolism inhibitors. **A** Matrigel tube formation assay. **B** Transwell. *P < 0.05, **P < 0.01. **Fig. S2**: miR-590-5p/Tiam1 regulated PC progression. **A** Matrigel tube formation assay. **B** Transwell. *P < 0.05, **P < 0.01. **Fig. S3**: Statistical analysis. **Fig. S4**: Statistical analysis. **Fig. S5**: Statistical analysis. **Fig. S6**: Statistical analysis. **Fig. S7**: Statistical analysis. **Fig. S8**: Statistical analysis. **Table S1**: Antibodies used in this work. **Table S2**: Reagents used in this work.

## Data Availability

The datasets used and/or analyzed during the current study are recorded in electronic laboratory notebook and available from the corresponding author upon reasonable request.

## Abbreviations

Tiam1: T lymphoma invasion and metastasis 1
PC: Pancreatic cancer
EdU: 5-Ethynyl-2′-deoxyuridine
qRT-PCR: Quantitative real time polymerase chain reaction
CoIP: Co-immunoprecipitation
SLC2A3: Glucose transporter protein 3
OS: Overall survival
EMT: Epithelial–mesenchymal transition
YAP: Yes-associated protein
TAZ: Transcriptional coactivator with PDZ-binding motif
Rac1: Ras-related C3 botulinum toxin substrate 1
OC: Ovarian cancer
BCa: Bladder cancer
GC: Gastric cancer
BC: Breast cancer
ATCC: American Type Culture Collection
DMEM: Dulbecco’s Modified Eagle Medium
FBS: Fetal Bovine Serum
WB: Western Blot
RLB: RIPA Lysis Buffer
PBS: Phosphate-buffered saline
ECL: Enhanced chemiluminescence
IHC: Immunohistochemistry
DAB: Double antibody
HR: Hazard ratio
CI: Class interval
LN: Lymph node
MTT: 3-[4,5-Dimethylthiazol-2-yl]-2,5 diphenyl tetrazolium bromide
IF: Immunofluorescence
CAM: Chorioallantoic membrane
HUVECs: Human umbilical vein endothelial cells
ATP: Adenosine triphosphate
2-DG: 2-Deoxy-d-glucose
3BrPA: 3-Bromopyruvate
CHX: Cycloheximide
MUT: Mutated-type
WT: Wild-type
