# Supplementary material for: miR-590-5p/Tiam1-mediated glucose metabolism promotes malignant evolution of pancreatic cancer by regulating SLC2A3 stability
Source: Cancer Cell Int. 2023 Nov 28;23:301. doi: 10.1186/s12935-023-03159-3 (PMC10685474; doi:10.1186/s12935-023-03159-3)
Supplement: Supplementary file 1 — Additional file 1. Fig. S1: Inhibition of Tiam1 expression in PC by glucose metabolism inhibitors. A Matrigel tube formation assay. B Transwell. *P < 0.05, **P < 0.01. Fig. S2: miR-590-5p/Tiam1 regulated PC progression. A Matrigel tube formation assay. B Transwell. *P < 0.05, **P < 0.01. Fig. S3: Statistical analysis. Fig. S4: Statistical analysis. Fig. S5: Statistical analysis. Fig. S6: Statistical analysis. Fig. S7: Statistical analysis. Fig. S8: Statistical analysis. Table S1: Antibodies used in this work. Table S2: Reagents used in this work. [file 12935_2023_3159_MOESM1_ESM.docx]

**Materials and methods**

**Cell viability assay**

MIAPaca-2 and Bxpc-3 Cells were plated in 96-well plates at a density of 5×10^3^ cells/well in DMEM with FBS for 24h, 48h, 72h and 96h. Cell viability was determined by the MTT solvent (Sigma). On the second day, add 100 µL MTT solution to each well for 4 hours before adding 100 µL DMSO. Finally, we measured the absorbance at 570nm and further evaluated number of cell proliferation for statistical analysis. All MTT assay results were presented as the means ± SD of three independent experiments.

**Colony formation experiment**

MIAPaca-2 and Bxpc-3 Cells were routinely digested and inoculated in 6-well plates (NEST, China) at a concentration of 2000 cells per well. Observe the growth status of cells every 2-3 days, clean cells and replace new cell culture medium. After culturing for about 14 days, the cells were fixed with 4% paraformaldehyde (YEASEN, USA) 15 min and stained with hematoxylin (solarbio, China). Perform statistical analysis using Image J and GraphPad Prism 9.0 software, the above experiments were repeated three times.

**5-ethynyl-29-deoxyuridine (EdU) assay**

When PC cells fuse to over 90%, wash the MIAPaca-2 and Bxpc-3 cells with PBS, and then perform routine digestion treatment. The cells were pulsed with A solvent 50μM 5-ethynyl-29-deoxyuridine (EdU kit, RiboBio, Guangzhou, China) for 2h, then fixed in 2% paraformaldehyde, and EdU detection was performed according to the manufacturer's instructions. Finally, we take pictures under the microscope and the above experiments were repeated three times.

**Wound healing assay**

After cell digestion, lay MIAPaca-2 and Bxpc-3 cells in a 6-well plate and conduct wound healing experiments after the cells adhere to the wall. Take a 200μL pipette and draw it from one side of the hole to the opposite side. Then, PBS was used for three times of cleaning and cell culture medium was added. Cell wound healing images were taken under a fluorescence microscope at 0h, 12h, 24h and 48 h. The above experiments were repeated three times and analyzed and statistically using software such as Image J and GraphPad Prism 9.0 software.

**Immunofluorescence**

After digestion PC cells, cells were inoculated onto glass cover slides. Cells grew to 70%, they were washed with PBS, fixed with 4% paraformaldehyde, and washed with 0.5% Triton X-100. Block the cells with 3% bovine serum albumin (A8020, Solarbio, Beijing, China) and incubate them overnight with the first antibody at 4°C. The next day, PBS washed the diluent of the first antibody and incubated the fluorescent second antibody. After 30 minutes of Hoechst staining, the film was sealed and all experiments were repeated three times. Finally, fluorescence microscopy was used for photography.

**Data availability**

The mRNA level of Tiam1 in tumor tissue and normal tissue was analyzed through http://ualcan.path.uab.edu, <http://gepia.cancer-pku.cn,> https://[www.oncomine.org/](http://www.oncomine.org/) and <https://sangerbox.org> [20]. Tiam1 survival analysis in PC has been determined on the website (http://kmplot.com/analysis/index). Predicting and analyzing potential miRNA targets through <http://www.targetscan.org,> <http://mirdb.org,> [https://starbase.sysu.edu.cn](https://starbase.sysu.edu.cn,) and http: //www.mircode.org/index [21].

**RNA extraction**

Total RNA was extracted from the PAAD cell using TRIzol® Reagent according the manufacturer’s instructions (Magen). RNA samples were detected based on the A260/A280 absorbance ratio with a Nanodrop ND-2000 system (Thermo Scientific, USA), and the RIN of RNA was determined by an Agilent Bioanalyzer 4150 system (Agilent Technologies, CA, USA). Sequencing Paired-end libraries were prepared using a ABclonal mRNA-seq Lib Prep Kit (ABclonal, China) following the manufacturer’s instructions. The mRNA was purified from 1 μg total RNA using oligo (dT) magnetic beads followed by fragmentation carried out using divalent cations at elevated temperatures in ABclonal First Strand Synthesis Reaction Buffer.

**RNA Sequencing**

First-strand cDNAs were synthesized with random hexamer primers and Reverse Transcriptase (RNase H) using mRNA fragments as templates, followed by second-strand cDNA synthesis using DNA polymerase I, RNAseH, buffer, and dNTPs. Adaptor-ligated cDNA were used for PCR amplification. PCR products were purified (AMPure XP system) and library quality was assessed on an Agilent Bioanalyzer 4150 system. Finally, the library preparations were sequenced on an Illumina. The above experiment was completed at APTBIO company.

**Supplementary Figure1**


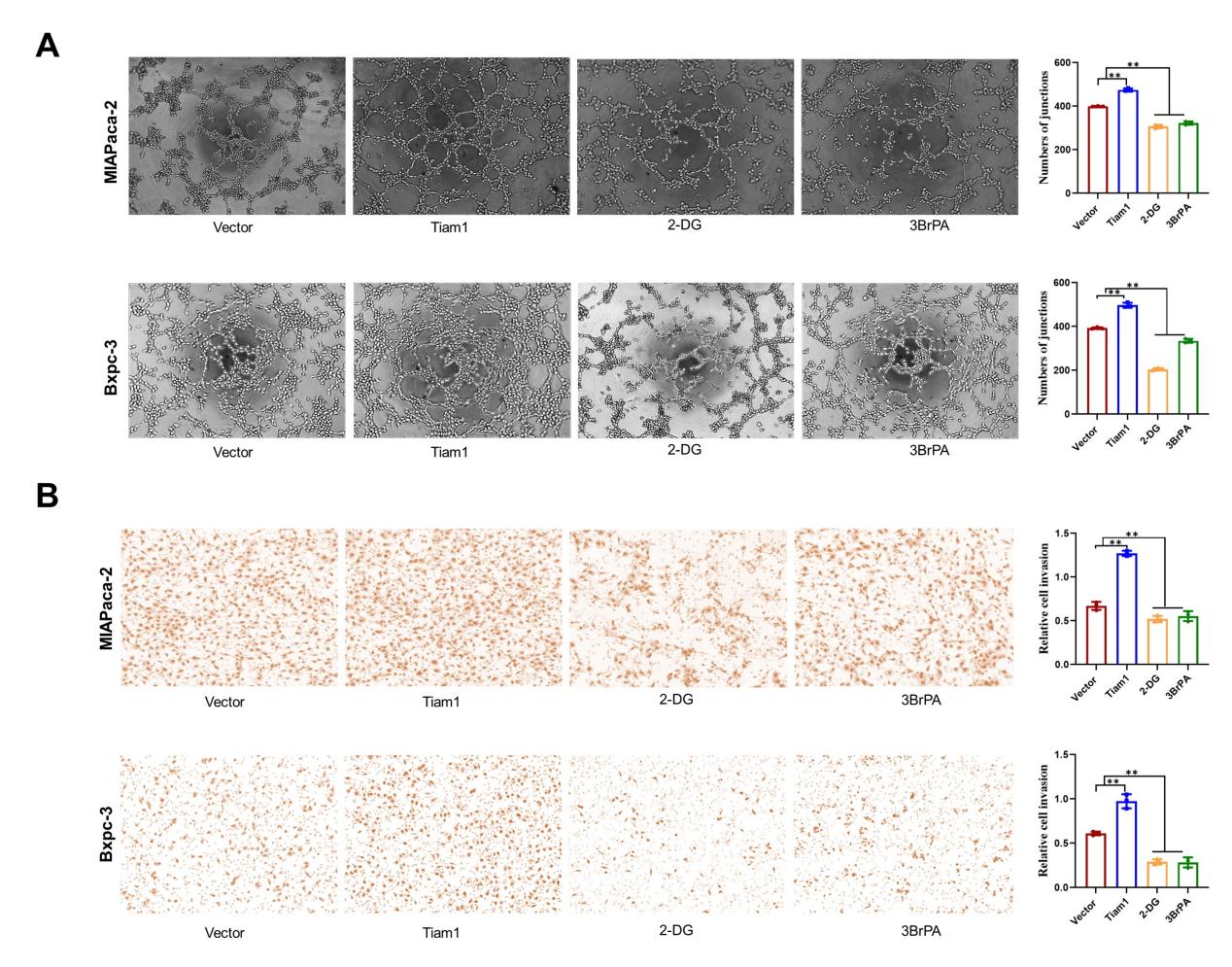


**Supplementary Figure1. Inhibition of Tiam1 expression in PC by glucose metabolism inhibitors.** **(A)** Matrigel tube formation assay. **(B)** Transwell. **P*< 0.05, ***P*< 0.01.

**Supplementary Figure2**


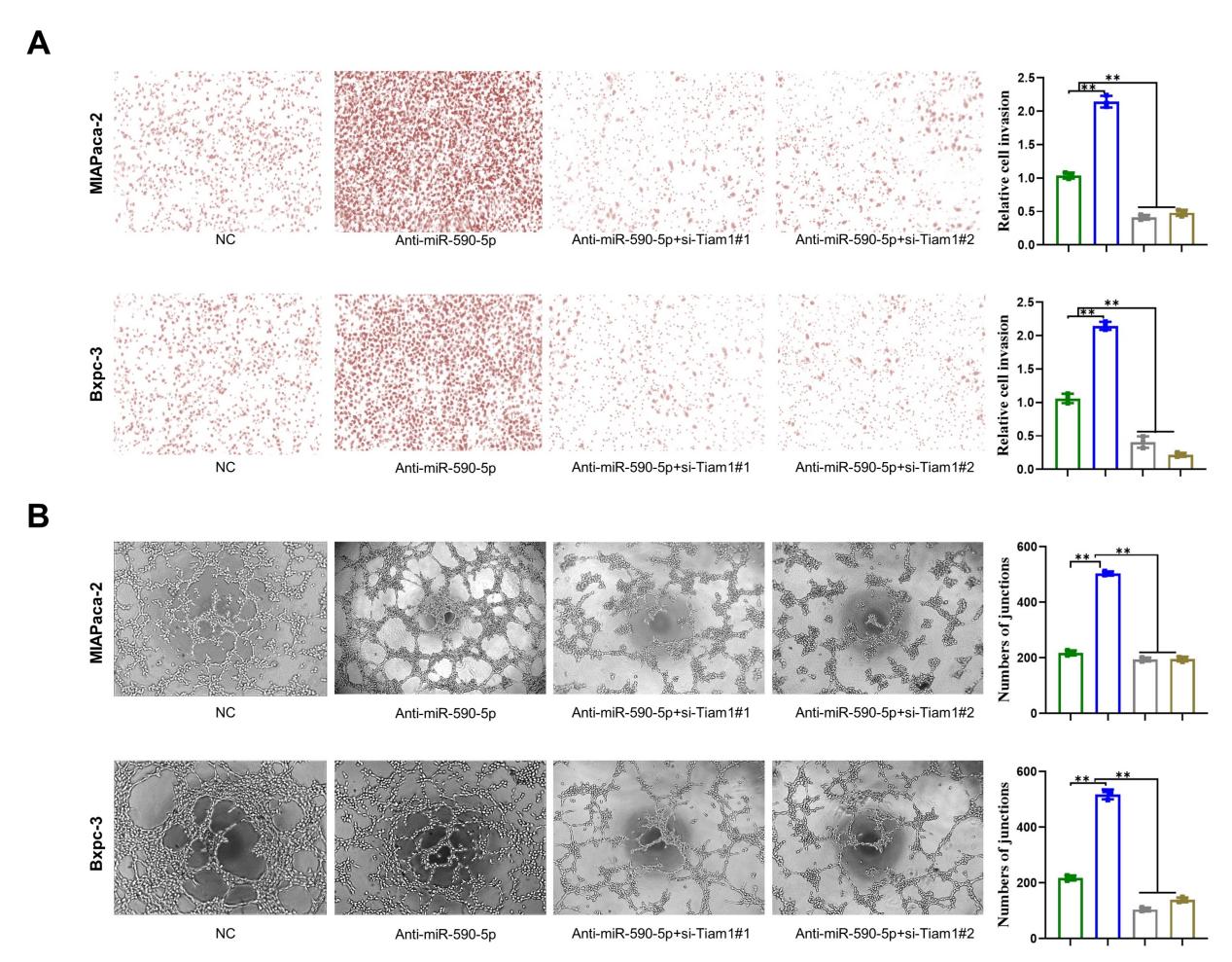


**Supplementary Figure2. miR-590-5p/Tiam1 regulated PC progression. (A)** Matrigel tube formation assay. **(B)** Transwell. **P*< 0.05, ***P*< 0.01.

**Supplementary Figure 3. Statistical analysis**

**
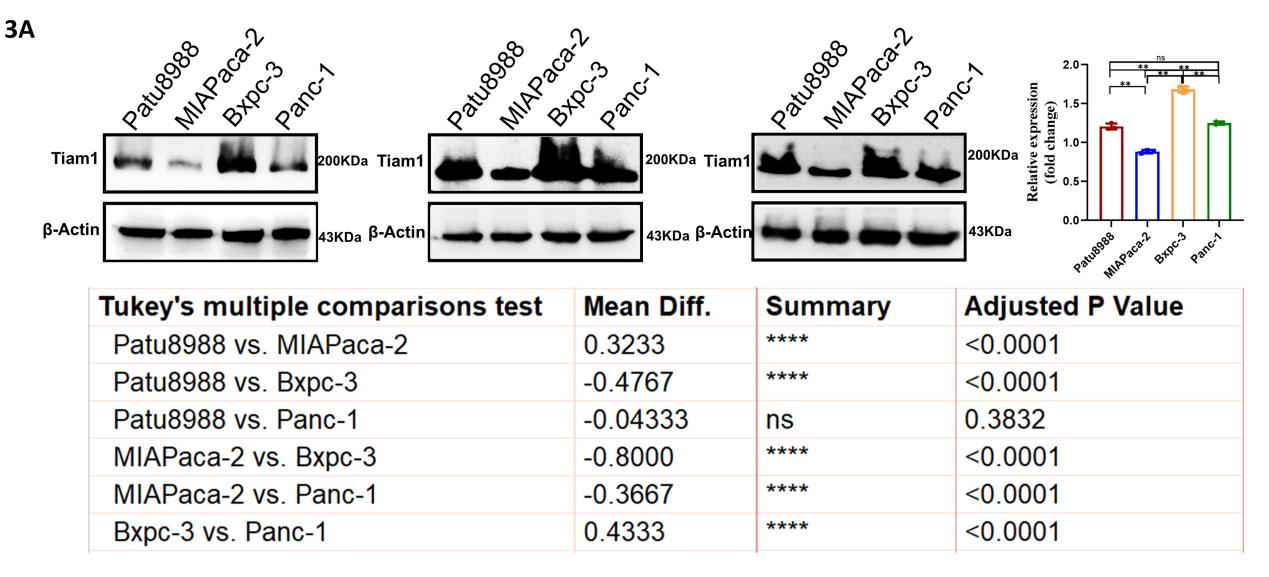
**

**Supplementary Figure 4. Statistical analysis**

**
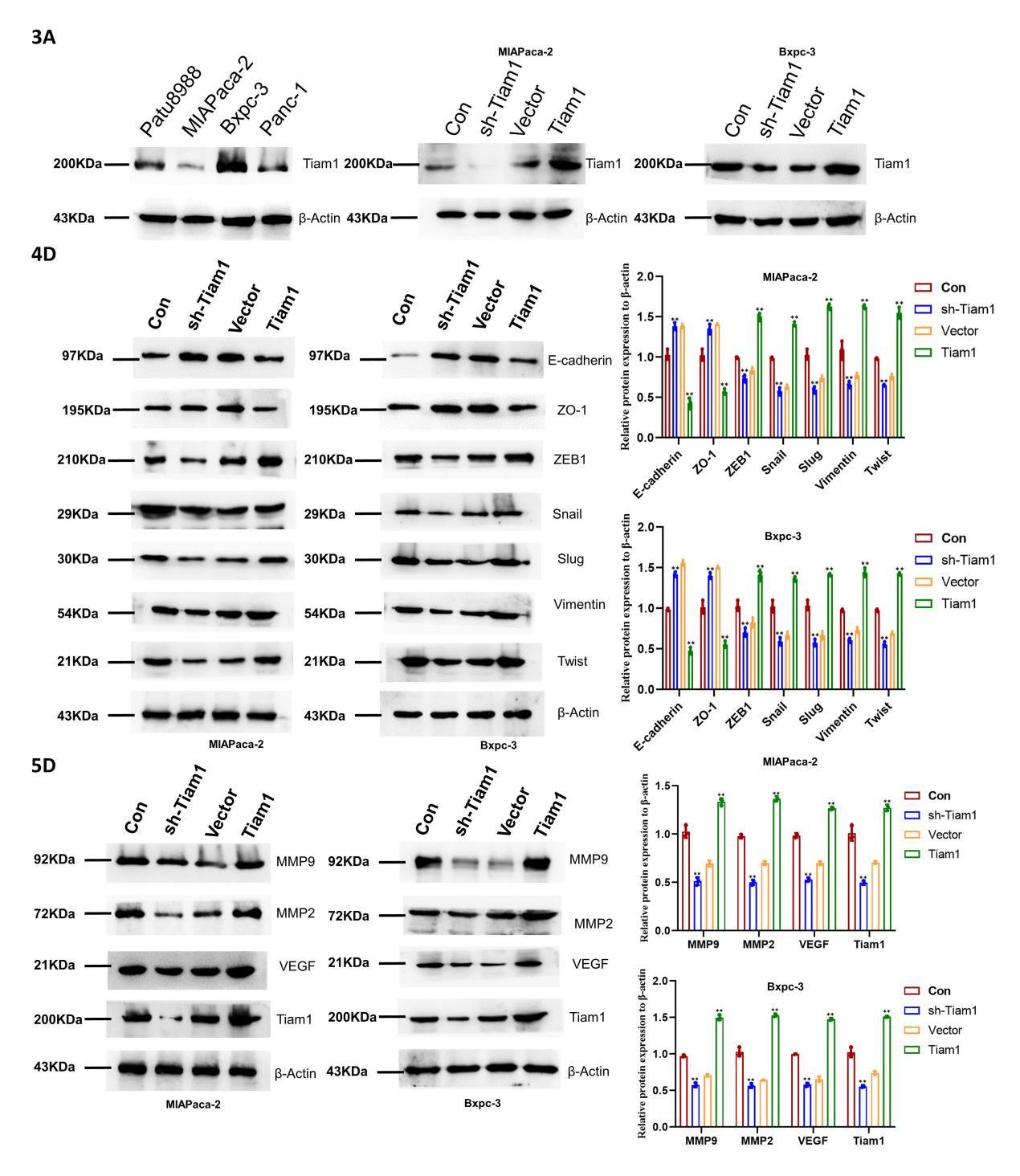
**

**Supplementary Figure 5. Statistical analysis**

**
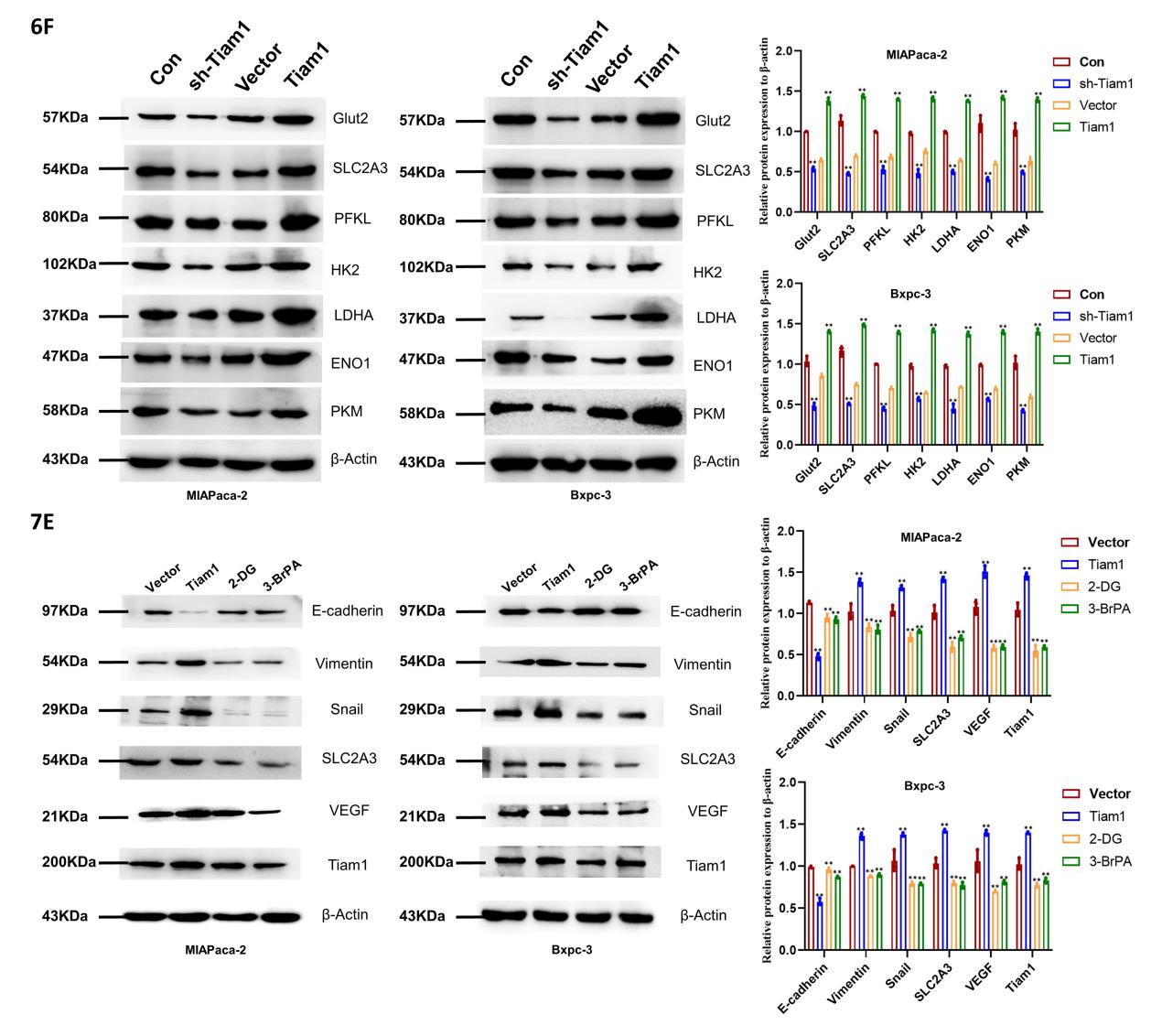
**

**Supplementary Figure 6. Statistical analysis**

**
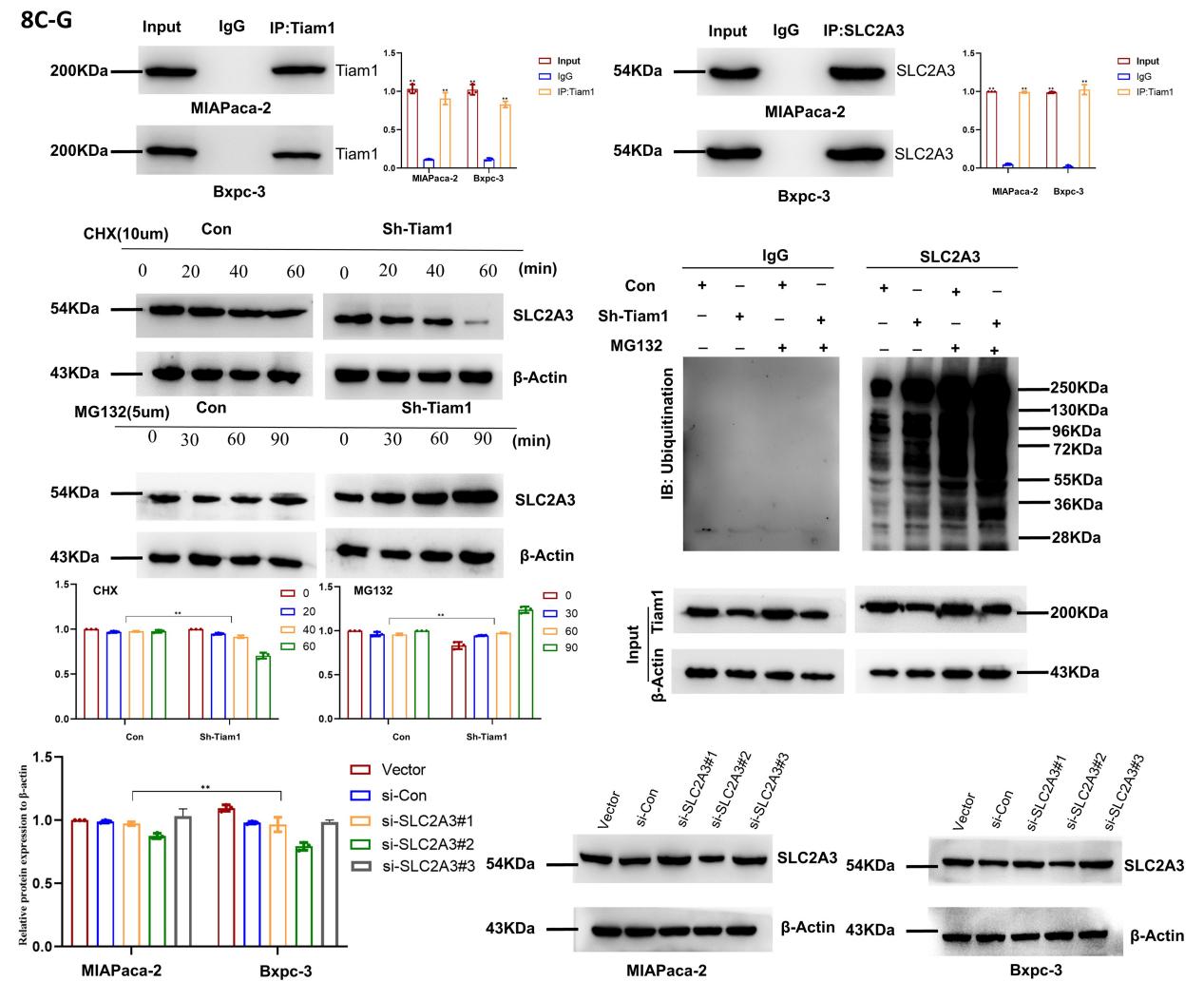
**

**Supplementary Figure 7. Statistical analysis**

**
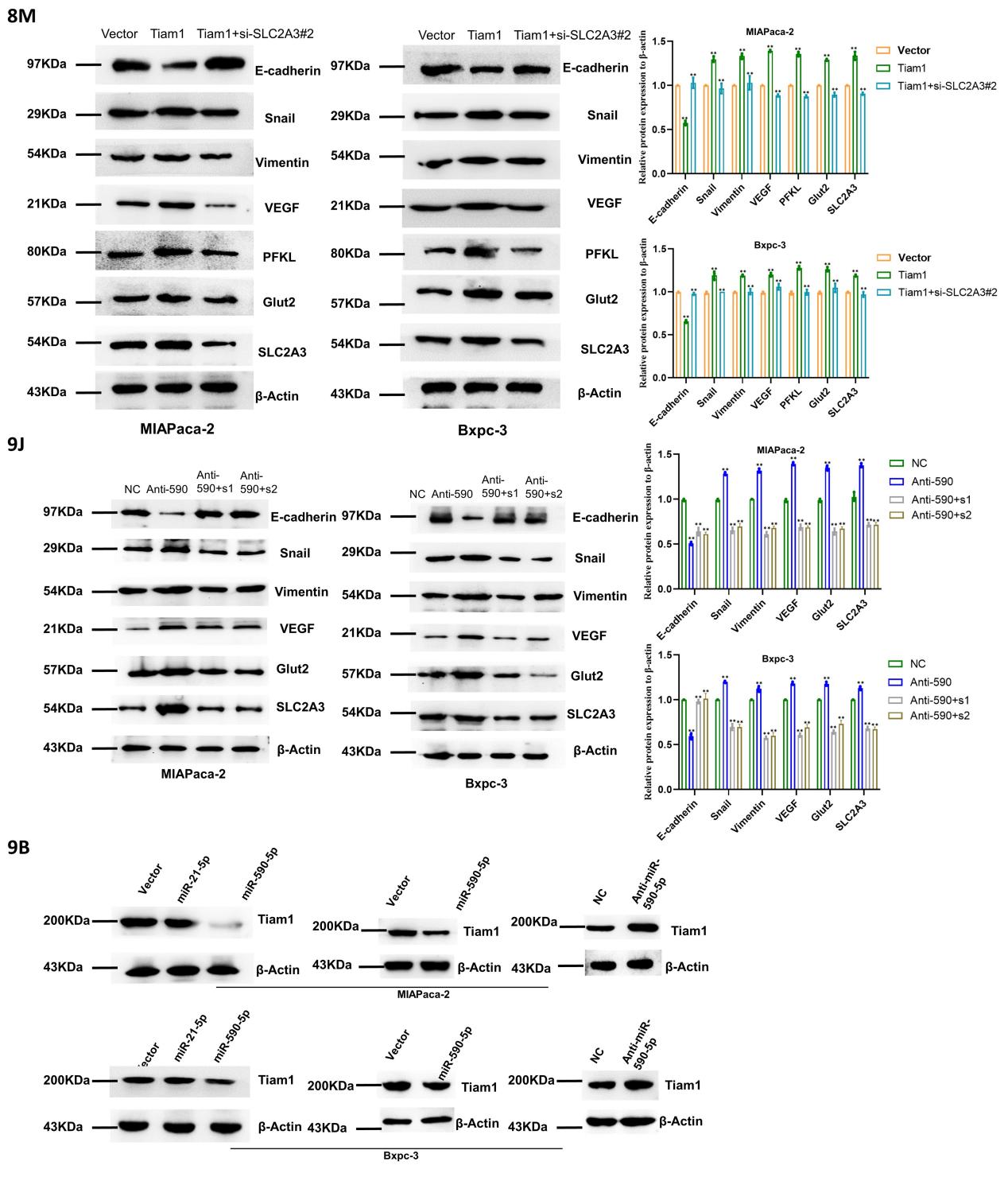
**

**Supplementary Figure 8. Statistical analysis**


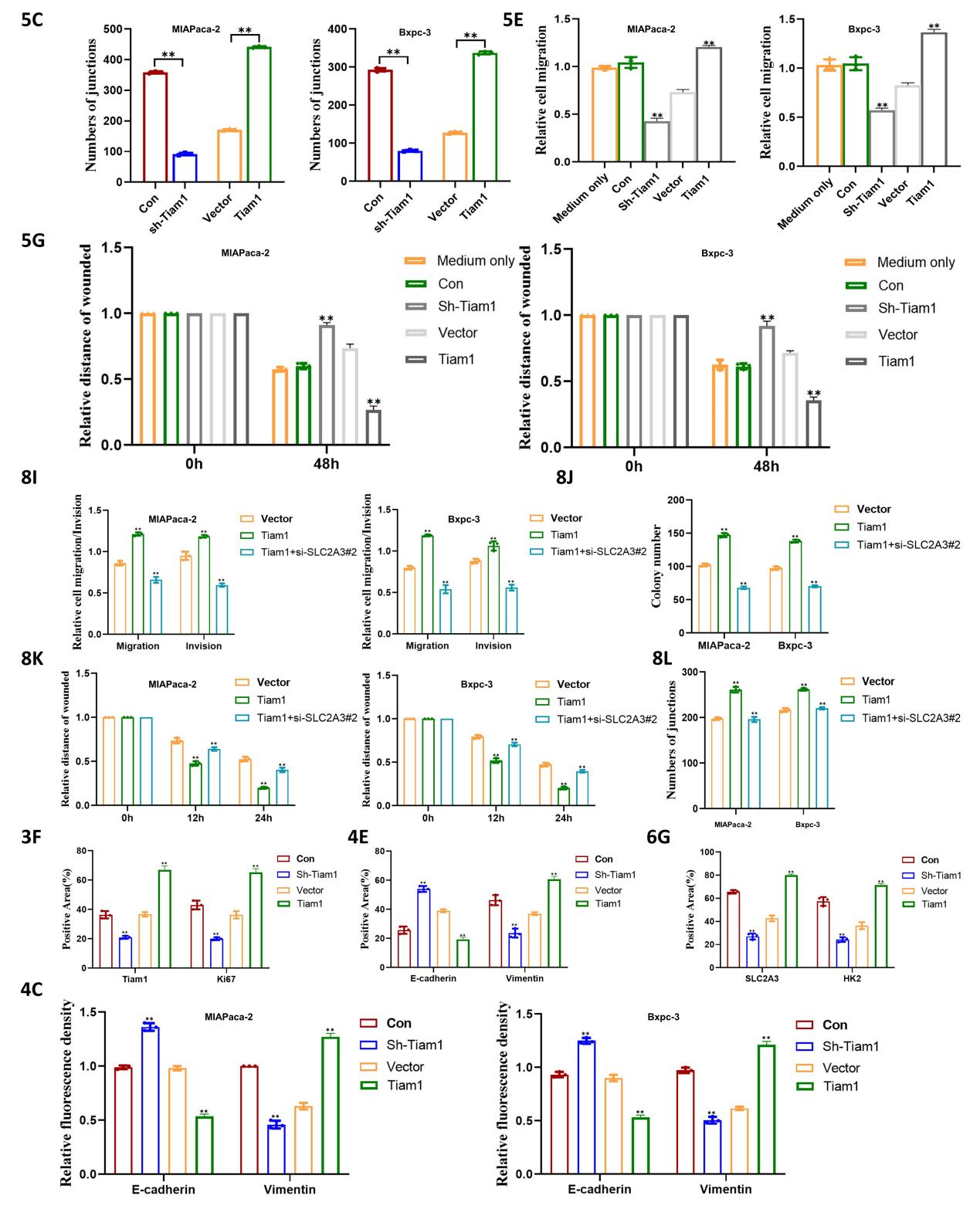


**Supplementary Table 1 Antibodies used in this work**

| Antibodies | Manufacturer | Number | Usage |
| --- | --- | --- | --- |
| Tiam1 | Santa-Cruz | sc-393315 | WB, IP, IF, ELISA |
| β-Actin | CWBIO | CW0096 | WB |
| E-Cadherin | Abcam | Ab40772 | Flow Cyt (Intra), ICC/IF, mIHC, IHC-P, WB |
| ZO-1 | Abcam | Ab264897 | WB, Sanger Sequencing |
| ZEB1 | Proteintech | 21544-1-AP | chIP, CoIP, IF, IHC, IP, WB |
| Snail | Proteintech | 13099-1-AP | ChIP, CoIP, IF, IP, WB |
| Slug | Proteintech | 12129-1-AP | IF, IHC, WB |
| Vimentin | Abcam | ab92547 | Flow Cyt (Intra), ICC/IF, WB, IHC-P, mIHC |
| Twist | Proteintech | 25465-1-AP | ChIP, CoIP, IF, IHC, IP, WB |
| MMP9 | Santa-Cruz | sc-393859 | WB, IP, IF, IHC(P) , ELISA |
| MMP2 | Santa-Cruz | sc-13594 | WB, IP, IF |
| VEGF | Santa-Cruz | sc-7269 | WB, IP, IF,IHC(P) |
| Glut2 | Proteintech | 20436-1-AP | IF, IHC, WB |
| SLC2A3 | Proteintech | 20403-1-AP | IF, IHC, WB |
| PFKL | Santa-Cruz | sc-393713 | WB, IP, IF, ELISA |
| HK2 | Proteintech | 22029-1-AP | CoIP, IF, IHC, IP, WB |
| LDHA | Proteintech | 19987-1-AP | CoIP, IF, IHC, IP, RIP, WB |
| ENO1 | Proteintech | 11204-1-AP | CoIP, IF, IHC, IP, WB |
| PKM | Proteintech | 15822-1-AP | ChIP, CoIP, IF, IHC, IP, WB |
| Ki67 | Proteintech | 27309-1-AP | IF, IHC |
| CHX | Santa-Cruz | CAS 66-81-9 | It inhibits the synthesis of proteins and macromolecules |
| MG-132 | Santa Cruz | A2585 | Proteasome inhibitor |
| Ub（P4D1） | Santa-Cruz | sc-8017 | WB, IP, IF, IHC(P), FCM, ELISA |

**Supplementary Table 2 Reagents used in this work**

| Reagents | Manufacturer | Company |
| --- | --- | --- |
| DMEM cell liquid culture medium | USA | GIBCO |
| Opti-MEM liquid culture medium | USA | GIBCO |
| fetal bovine serum(FBS) | USA | GIBCO |
| Penicillin/Streptomycin | USA | GIBCO |
| trypsin | USA | GIBCO |
| Matrigel | USA | Corning company |
| BCA Protein Quantitative | China | Cwbio |
| SDS gel preparation kit | China | Cwbio |
| Hypersensitivity ExPlus ECL Chemiluminescence Kit | China | ZOMANBIO |
| skim milk | USA | BD company |
| EdU reagent kit | China | RiboBio |
| RNA extraction reagent | China | ZOMANBIO |
| Bulge-LoopTM miRNA qRT-PCR Primer | China | RiboBio |
| UltraSYBR Mixture (Low ROX) | China | [Beyotime](https://www.baidu.com/link?url=IIImGs_M9DixvZZrE3eKwB3k0zIymEO6Z4yOTQwlifELjfSj47aBXzEXYiIz4K7C&wd=&eqid=ba0c42a700062eb8000000026549f4be) |
| Lipofectamine 3000 | USA | ThermoFisher |
| Protein A/G agarose beads | USA | Santa-Cruz |
| DMSO | China | solarbio |
| MTT | USA | Sigma |
| Immobilon®-P PVDF membrane | Ireland | Merck Millipore Ltd |
| coverslip | China | [Beyotime](https://www.baidu.com/link?url=IIImGs_M9DixvZZrE3eKwB3k0zIymEO6Z4yOTQwlifELjfSj47aBXzEXYiIz4K7C&wd=&eqid=ba0c42a700062eb8000000026549f4be) |
| Western semi-dry Transfer buffer | China | [Beyotime](https://www.baidu.com/link?url=IIImGs_M9DixvZZrE3eKwB3k0zIymEO6Z4yOTQwlifELjfSj47aBXzEXYiIz4K7C&wd=&eqid=ba0c42a700062eb8000000026549f4be) |
| Hematoxylin | China | solarbio |
| Alexa Fluor Anti-Mouse/Rabbit IgG （H+L） | USA | ThermoFisher |
| neutral balsam | China | Biosharp |
| Glucose uptake colorimetric determination kit | China | Rongsheng Biotechnology Co., Ltd. |
| lactic acid colorimetric determination kit | China | Nanjing Jiancheng Institute of Bioengineering |
| ATP kit | China | Nanjing Jiancheng Institute of Bioengineering |
